# Uranium Isotope Evidence for Persistent Global Ocean Anoxia Across the Ediacaran–Cambrian Transition in Siberia

**DOI:** 10.1111/gbi.70066

**Published:** 2026-09-26

**Authors:** Andrew D. Doerrler, Tian Gan, Geoffrey J. Gilleaudeau, Matthew G. Pedersen, Natalia Bykova, Vasiliy Marusin, Natalia A. Ivanova, Boris Kochnev, Dimitriy V. Grazhdankin, Karel Kletetschka, Alan J. Kaufman

**Affiliations:** ^1^ Department of Geological, Environmental, and Planetary Sciences University of Maryland College Park Maryland USA; ^2^ Department of Atmospheric, Oceanic, and Earth Sciences George Mason University Fairfax Virginia USA; ^3^ Department of Geological Sciences University of Missouri Columbia Missouri USA; ^4^ Trofimuk Institute of Petroleum Geology and Geophysics Novosibirsk Russian Federation; ^5^ Department of Geology and Geophysics Novosibirsk State University Novosibirsk Russian Federation; ^6^ Siberian Research Institute of Geology, Geophysics, and Mineral Raw Materials Novosibirsk Russian Federation; ^7^ State Key Laboratory of Continental Evolution and Early Life, Shaaxi Key Laboratory of Early Life and Environments, Department of Geology Northwest University Xi'an China; ^8^ Earth System Science Interdisciplinary Center University of Maryland College Park Maryland USA

**Keywords:** BACE, carbonate, Ediacaran–Cambrian transition, ocean redox, Siberia, uranium isotopes

## Abstract

The enigmatic Ediacara biota represents Earth's earliest example of macroscopic, complex life, yet the driving forces promoting their mid‐Ediacaran evolution and subsequent extinction across the Ediacaran–Cambrian boundary remain speculative. If these were true animals requiring oxygen for aerobic respiration, their rise has often been linked to dramatic changes in the redox state of seawater. Their disappearance from the fossil record—whether gradual or abrupt—and replacement with Cambrian animals has similarly been linked to redox fluctuations, suggesting an environmental driver, or alternatively to biological factors such as predation of the predominantly soft‐bodied organisms and bioturbation that disrupted the microbial mats in which they were intimately associated. Insofar as Ediacara extinction is linked to a major perturbation of the global carbon cycle (the BAsal Cambrian carbon isotope Excursion or BACE), an environmental driver seems likely. On the one hand, their demise might reflect the spread of seawater anoxia if these organisms had aerobic metabolisms, but on the other, widespread ocean oxygenation may have been the proximal cause of their extinction and the BACE event if the biota was tolerant and even thrived under low‐oxygen conditions. To test between the two alternative redox scenarios, we present a suite of geochemical data including uranium isotopes (δ^238^U) from carbonate and evaporite‐rich successions that span the BACE on the Siberian Platform. Like conditions before and after the event, consistently low δ^238^U values from both sections (median reconstructed seawater δ^238^U = −0.74‰ to −0.79‰) suggest that widespread ocean anoxia persisted across the Ediacaran–Cambrian transition. The integrated data indicate that neither a rise nor fall of seawater oxidation state led to the demise of the Ediacara biota. Lacking evidence for a redox driver, we hypothesize that sea‐level regression and the widespread development of evaporative conditions in shallow‐water environments negatively impacted the largely immobile, diffusion‐dependent, and potentially stenothermal Ediacara biota by subaerial exposure, dehydration through osmosis, and/or large temperature variations in shallow water. Thereafter, normal salinity and subtle rises in shallow ocean O_2_ levels in concert with tectonic and ecological factors could have driven the Cambrian radiation in oceans that remained O_2_‐depleted well into the Paleozoic Era.

## 1 Introduction

Earth's first sustained experiment in complex macroscopic life occurred during the Ediacaran Period (635.2–538.8 Ma; Narbonne et al. 2012; Xiao et al. 2016; Linnemann et al. 2019). In the fossil record, these largely sessile and soft‐bodied organisms are preserved primarily as impressions, casts, or molds in fine‐grained siliciclastic rocks (Narbonne 2005). Due to their unusual taphonomic mode and bizarre morphologies, the metabolic requirements of these organisms remain obscured, although their fractal patterns and large surface areas suggest that diffusion was important (Narbonne 2005; Laflamme et al. 2013; Grazhdankin 2014). This possibility has led to interpretations of feeding strategies that include osmotrophy of dissolved organic matter and oxygen for heterotrophic respiration, or of hydrogen sulfide that fueled internal autotrophy by symbiotic sulfur‐oxidizing bacteria (Laflamme et al. 2009; Kaufman et al. 2019; McIlroy et al. 2021). Given these contrasting views of Ediacaran lifestyles, the kill mechanism(s) for the soft‐bodied biota across the Ediacaran–Cambrian boundary fall into several camps (Laflamme et al. 2013). The biotic replacement hypothesis suggests that the advent of predation and bioturbation were the proximal causes for the gradual demise of the Ediacara biota insofar as they were outcompeted by Cambrian animals for suitable substrates (Bottjer et al. 2000; Seilacher et al. 2005). In contrast, the environmental hypothesis suggests that the Ediacara biota was quickly eliminated due to some perturbation of global seawater chemistry thereby allowing subsequent Cambrian organisms to fill vacated niches and create new ones through ecosystem engineering (Laflamme et al. 2013), although the recently discovered terminal Ediacaran Jiangchuan Biota (SW China) records both Ediacaran organisms and early bilaterians living in the same ecological community (Li et al. 2026).

**FIGURE 1:**
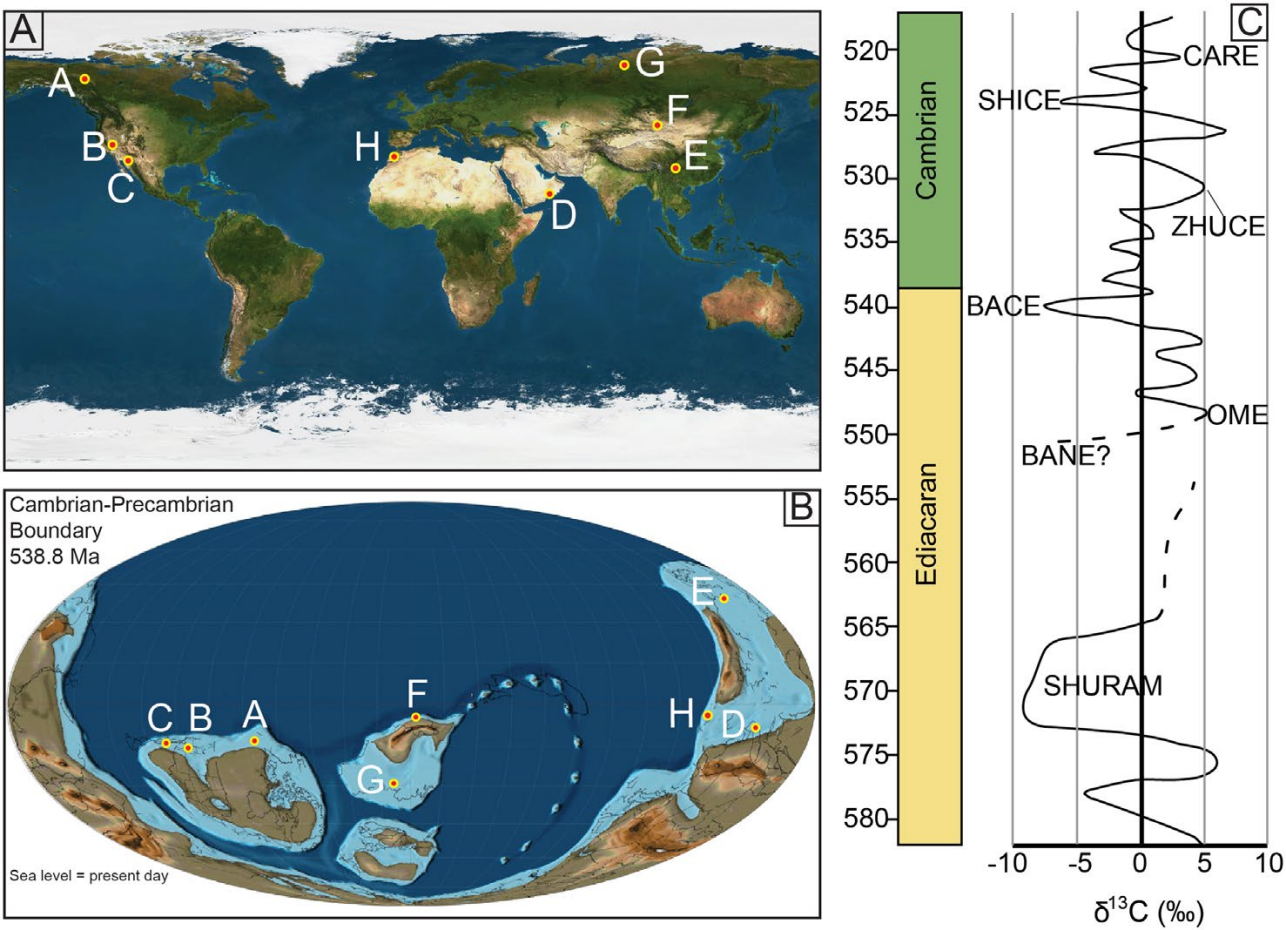
Modern global map, paleogeographic map, and global δ^13^C curve for the Ediacaran–Cambrian transition. A–B, BACE locations shown for both modern (A) and Precambrian–Cambrian boundary continental configurations (B) (paleogeographic base map after Scotese, 2016). The locations of letters A–G on the map correspond with sections globally where the BACE is recorded: (A) Northwest Canada; (B) Western United States; (C) Mexico; (D) Oman; (E) South China; (F) Mongolia; (G) Siberia (see Topper et al. 2022 for summary); (H) Morocco (Maloof et al. 2010). C, Global δ^13^C chemostratigraphy of the Ediacaran–Cambrian transition (modified from Bowyer et al. 2022).

Lending support to an environmental driver, the disappearance of the Ediacara biota broadly coincides with a significant geochemical perturbation—a global negative carbonate carbon isotope (δ^13^C_carb_) anomaly with nadir values in some sections near −10‰ known as the BAsal Cambrian carbon isotope Excursion or BACE (Figure 1; Zhu et al. 2006). This biogeochemical event—similar in magnitude to the terminal Ediacaran Shuram Excursion—has been broadly recognized in carbonates of the Northwest Territories of Canada (Narbonne et al. 1994; Kaufman et al. 1997), the western United States (Corsetti and Kaufman 1994; Smith, Nelson, et al. 2016), Mexico (Sour‐Tovar et al. 2007; Loyd et al. 2012; Hodgin et al. 2020), Morocco (Maloof et al. 2005; Maloof et al. 2010), Oman (Amthor et al. 2003; Bowring et al. 2007), Iran (Kimura et al. 1997), China (Li and Xiao 2004; Ishikawa et al. 2008; Li et al. 2009; Jiang et al. 2012; Steiner et al. 2020), Mongolia (Bold et al. 2016; Smith, Macdonald, et al. 2016; Topper et al. 2022), Kazakhstan (Stammeier et al. 2019), India (Kaufman et al. 2006), and Siberia (Knoll et al. 1995; Kaufman et al. 1996; Pelechaty et al. 1996; Bartley et al. 1998; Kouchinsky et al. 2007).

Constraining the timing of the BACE event requires integration of the known stratigraphic record of trace and body fossils of Ediacaran and Cambrian aspect, as well as radiometric ages provided by U–Pb analyses of zircons from volcanic ash deposits and of detrital zircons in siliciclastic rocks. Most impressions of the soft‐bodied Ediacara biota are restricted to pre‐BACE strata with the rare exception of frondose organisms similar to *Swartpuntia* in lower Cambrian successions of South Australia (Jensen et al. 1998) and the western USA (Hagadorn et al. 2000), locally abundant *Stramatoveris* in the lower Cambrian Chengjiang Formation of South China (Hoyal Cuthill and Han 2018), and the middle Cambrian *Thaumaptilon* from the Burgess Shale (Morris 1993; Hoyal Cuthill 2022). In contrast, the assemblage of ichnofossils that define the base of the Cambrian System (Landing 1994), including *Trepichtnus pedum*, a distinctive branched trace fossil of bilaterian origin, is found in siliciclastic strata that post‐date the BACE, which is preserved in carbonates (Nelson et al. 2023; Zhang et al. 2024). The last occurrence of the worm‐like cloudiinids with their biomineralized funnel‐in‐funnel carbonate shells appears to precede the BACE in most localities (but see Topper et al. 2022, who report the presumed cloudinomorph *Zuunia chimidtsereni* during the BACE in Mongolia at the top of the Zuun‐Arts Formation and continuing upsection into the basal Bayangol Formation, as well as other rare examples of Cambrian cloudiinids; Yang et al. 2016; Zhu et al. 2017). Except for *Protohertzina anabarica* and *P. unguliformis* found in phosphatic carbonates near the nadir of the BACE in the Mongolian succession, no other biomineralized small shelly fossils (SSFs) of Cambrian aspect are currently known to co‐occur with the profound negative carbon cycle anomaly (Topper et al. 2022). In Oman, a volcanic ash with a U–Pb zircon age of 541.00 ± 0.13 Ma (Bowring et al. 2007) lies below a negative carbon isotope excursion within thick interbedded BACE carbonates and evaporites of the Ara Group, while in South China a volcanic ash above the BACE yielded a U–Pb zircon age of 535.2 ± 1.7 Ma (Zhu et al. 2009). Detrital zircon U–Pb radiometric age constraints for the BACE in Mexico provide a maximum depositional age (MDA) for the event that is younger than 539.40 ± 0.23 Ma (Hodgin et al. 2020), while in the western USA, the youngest population of detrital zircon grains from a siltstone bed within the lower Wood Canyon Formation just above the BACE (Smith, Nelson, et al. 2016) suggests that the carbon isotope anomaly is just slightly older than 532.84 ± 0.98 Ma (Nelson et al. 2023). Accepting the volcanic ash U–Pb ages in isolation, the BACE would lie somewhere in a six‐million‐year interval between 541 and 535 Ma, which, given the currently ratified age of the Ediacaran–Cambrian boundary (ca. 538.8 Ma; Linnemann et al. 2019), suggests that the BACE spans across this geological transition. On the other hand, if the MDAs of detrital zircons from Laurentia are used, the BACE would be constrained to the interval between ca. 539 and 533 Ma and thus be basal Fortunian in age.

The ultimate cause of the BACE and the mass extinction of the Ediacara biota remains uncertain, but changes in seawater redox state potentially played a role in both. It has long been hypothesized that a rise in oxygen to near 10% of PAL (present atmospheric level) was necessary to sustain the metabolic activities of large macroscopic animals (Knoll 1996). This paradigm was supported by early geochemical models of time‐series isotopic data from Neoproterozoic successions worldwide suggesting a geologically rapid rise of *p*O_2_ after the Marinoan ice age (Derry et al. 1992; Kaufman et al. 1993), which in part led to the concept of a Neoproterozoic Oxygenation Event or NOE (Shields‐Zhou and Och 2011). Supporting the NOE and its linkage to early macroscopic life, iron speciation analyses of siliciclastic mudstone and shale from a fossiliferous terminal Ediacaran succession in Newfoundland (Canfield et al. 2007) were interpreted as the result of the ventilation of deep subtidal environments that supported a biota far below the photic zone. However, a later global compilation of Ediacaran shale iron speciation data suggested that anoxic conditions more broadly characterized the period (Sperling et al. 2015), in addition to the persistence of crustal thallium isotope values in Ediacaran shales (Ostrander 2023), also suggesting widespread ocean anoxia. Interpreting a middle ground, a carbonate‐based iron speciation study in the upper Nama Group of Namibia suggested a more dynamic redox landscape that affected emergent terminal Ediacaran ecosystems (Wood et al. 2015). A large compilation of carbonate cerium (Ce) anomaly data suggests transient oxygenation of the shallow oceans broadly across the Ediacaran‐Cambrian boundary (Wallace et al. 2017), although iodine‐calcium (I/Ca) ratios in carbonates suggest minimal surface oxygenation during this interval (Lu et al. 2018).

As an alternative redox indicator, uranium isotope ratios in carbonates have emerged as a powerful geochemical proxy for global redox conditions, especially in the terminal Ediacaran and basal Cambrian intervals. Studies from South China, Namibia, and Siberia (Zhang et al. 2018; Tostevin et al. 2019; Cherry et al. 2022) all point to widespread anoxia in the stratigraphic window containing the Ediacara biota with some of the lowest δ^238^U values ever recorded. While one study from the Nama Group in Namibia (Tostevin et al. 2019) concludes that the global record of δ^238^U variations is consistent with dynamic redox conditions (as previously interpreted from iron speciation analyses; Wood et al. 2015), another from the Dengying Formation in South China (Zhang et al. 2018) proposes that increasing anoxia towards the Ediacaran–Cambrian boundary promoted the demise of the biota assumed to utilize aerobic respiration for their metabolic activities. A recent uranium isotope and cerium anomaly study of terminal Ediacaran carbonates in arctic Siberia proposed instead that the biota may have tolerated low‐oxygen conditions (Cherry et al. 2022) potentially relying on chemosymbionts that exploited redox gradients in low‐oxygen oceans (Kaufman et al. 2019). If correct, a rise in oceanic O_2_ could have been the driver for their extinction and replacement with the more energetic and O_2_‐demanding organisms of the Cambrian fauna. This view is consistent with the idea that complex multicellular life must have harnessed and internalized hypoxic conditions early in its history (Hammarlund 2020). Uranium isotope studies of the BACE interval have thus far been limited, but Chanchai et al. (2026) reported a lack of significant δ^238^U change in two BACE sections from southwestern Laurentia arguing against global ocean redox change across the Ediacaran–Cambrian transition.

**FIGURE 2:**
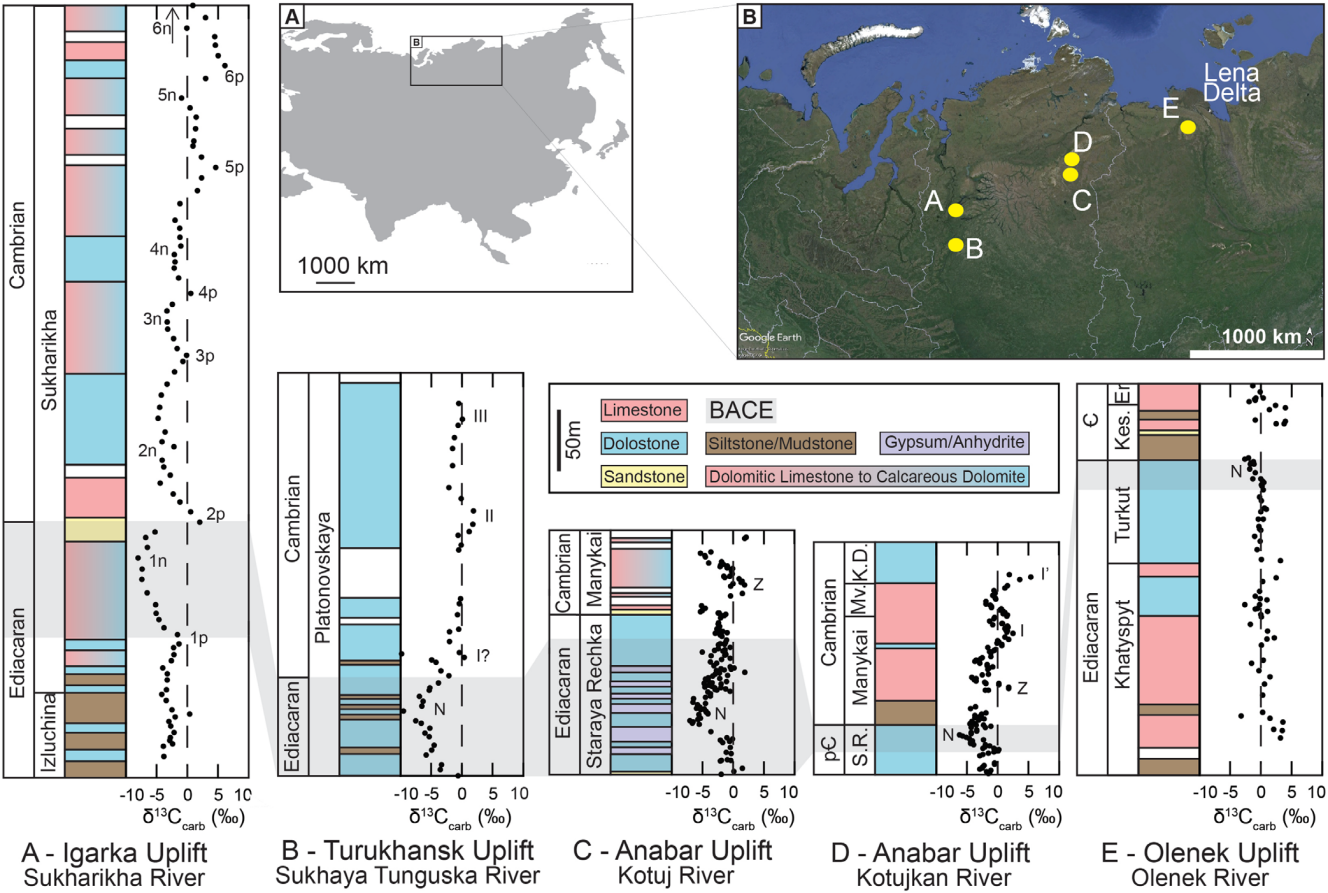
Regional correlation for the Ediacaran–Cambrian transition across the northern Siberian Platform. (A) Map of eastern Europe and Asia showing the location of panel (B). B, Locality map of northern Siberia, showing the locations of well‐studied Ediacaran–Cambrian successions in this region. The location of letters A–E on the map correspond with the stratigraphic columns and δ^13^C_carb_ curves below: (A) Igarka Uplift, Sukharikha River (Kouchinsky et al. 2007; Marusin et al. 2023); (B) Turukhansk Uplift, Sukhaya Tunguska River (Bartley et al. 1998); (C) Anabar Uplift, Kotuj River (this study); (D) Anabar Uplift, Kotujkan River (Knoll et al. 1995; Kaufman et al. 1996); (E) Olenek Uplift, Olenek River (Knoll et al. 1995; Pelechaty et al. 1996). S.R. = Staraya Rechka Formation, Mv. = Medvezhya Formation, K.D. = Kyndyn Dolostone, Kes. = Kessyusa Group, Er. = Erkeket Formation, pꞒ = Precambrian, Ꞓ = Cambrian. The gray band represents the BACE interval, which is denoted by 1n or N depending on the publication. The letters or numbers associated with other δ^13^C_carb_ excursions are based on the original publications. Note that the correlation of the BACE interval is speculative in some sections, and that alternative models for correlation do exist between these sections (see Marusin et al. 2022, 2025 and Section 2 for detailed discussion).

To further test the oxygen kill hypothesis and provide constraints on potential environmental drivers for the extinction of the Ediacara biota, we present new paleo‐redox data from three carbonate‐dominated successions in Siberia (Figure 2). Specifically, we provide carbonate uranium isotope (δ^238^U) and REE (Ce anomaly) data for the Kotuj and Sukharikha river sections and pyrite sulfur isotope (δ^34^S) data from the previously investigated hypostratotype along the Kotujkan River (Knoll et al. 1995; Kaufman et al. 1996). We additionally present ^87^Sr/^86^Sr data from the Sukharikha River succession, which highlights the well‐preserved nature of the samples and contributes an important new ^87^Sr/^86^Sr record for the Fortunian Stage. The carbonate δ^238^U proxy has emerged as a powerful tool for reconstructing the global redox state of ancient oceans, grounded in the preferential removal of ^238^U from seawater under anoxic conditions, leading to lower δ^238^U values in seawater (and thus carbonates) during periods of widespread ocean anoxia. Alternatively, periods of widespread ocean oxygenation, such as today, are predicted to result in higher δ^238^U values in seawater and carbonates. Thus, if the BACE were associated with an ocean oxygenation event, we would predict pronounced ^238^U enrichments to be recorded in our carbonate sections.

## 2 Geologic Background and Context

### 2.1 Geological Setting: Anabar Uplift

The Staraya Rechka and Manykai formations were examined at two locations in the western Anabar Uplift of the Siberian Platform along the Kotuj and Kotujkan (tributary of the Kotuj) rivers. Our northern exposure along the Kotujkan River (the hypostratotype) comprises 180 m of shallow‐marine carbonates and subordinate siliciclastics divided into three principal sequences represented by the Staraya Rechka, Manykai (named the Nemakit‐Daldyn Formation in Marusin et al. 2025), and Medvezhya formations (Knoll et al. 1995; Kaufman et al. 1996). Here, the Staraya Rechka Formation is approximately 40 m thick and separated from underlying carbonates and evaporites of the Mesoproterozoic Yusmastakh Formation by an angular unconformity (Khomentovsky 1990; Bartley et al. 2000). The Staraya Rechka Formation comprises a lithologically complex interleaving of dolostones (including stromatolitic, grainstone, and mudstone fabrics), sulfate evaporites, and siliciclastics representing a range of shallow subtidal and restricted peritidal depositional environments. Sulfate evaporites occur as nodules and lozenge‐shaped molds in dolomitic mudstone in the lower and upper parts of the formation. A regional disconformity between the Staraya Rechka and overlying Manykai formations—characterized by breccias and karstic solution features—is suggested to mark the beginning of the basal Cambrian Period (Savitsky 1978). The Manykai Formation, which is ~90 m thick in the northern exposure, includes a basal interval of mixed siliciclastic and carbonate rocks that grade upward into limestones and dolomitic limestones, including mudstone, grainstone, microbialaminite, and thrombolite or algal facies. The Manykai depositional sequence is subdivided into four aggradationally stacked parasequences separated by flooding surfaces and nine distinct beds (Khomentovsky and Trofimov 1980; Kaufman et al. 1996) with thrombolites and calcareous algae prominent in the lower interval of Bed V and throughout Bed IX (known as the Koril Member containing the branched calcareous algae *Korilophyton*; Luchinina 2009).

**FIGURE 3:**
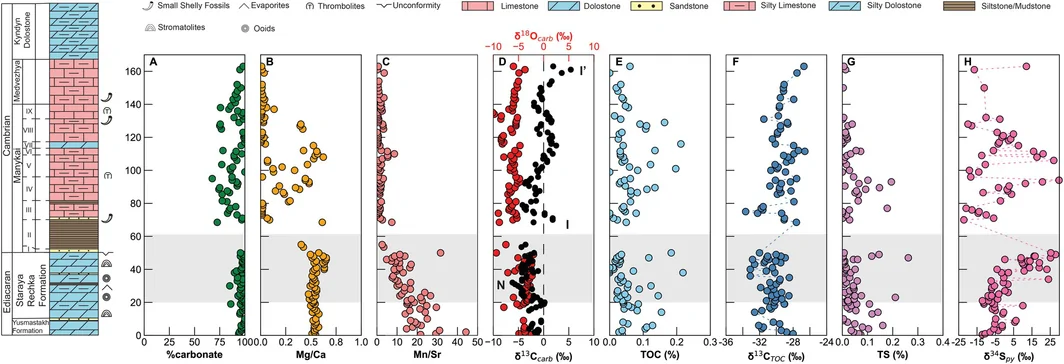
Stratigraphy and chemostratigraphy of the Staraya Rechka, Manykai, Medvezhya, and Kyndyn formations in the Kotujkan River section of the northern Anabar region previously investigated by Knoll et al. (1995) and Kaufman et al. (1996). (A) Percentage of carbonate in the samples. (B) Mg/Ca ratios. (C) Mn/Sr ratios. (D) δ^13^C_carb_ and δ^18^O_carb_ chemostratigraphy. δ^13^C_carb_ and δ^18^O_carb_ are referenced to V‐PDB. (E) Total organic carbon (TOC) in samples. (F) δ^13^C_TOC_ referenced to V‐PDB. (G) Total sulfur in samples. H, δ^34^S_py_ from acidified residues referenced to V‐CDT. The gray band represents the BACE interval. N denotes the BACE and I and I′ denote other δ^13^C_carb_ excursions based on the original publications.

**FIGURE 4:**
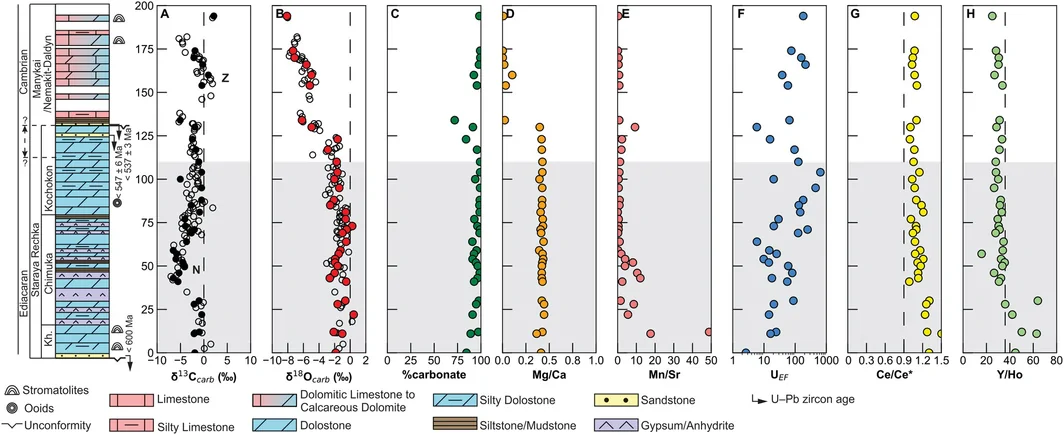
Stratigraphy and chemostratigraphy of the Staraya Rechka and Manykai formations in the southern Kotuj River section. A–B, δ^13^C_carb_ (A) and δ^18^O_carb_ (B) chemostratigraphy. δ^13^C_carb_ and δ^18^O_carb_ are referenced to V‐PDB. Closed circles represent samples analyzed for δ^238^U and elemental concentrations in this study. Open circles represent samples that were previously measured by Marusin et al. (2025). C, Percentage of carbonate in the samples. D, Mg/Ca ratios from the U leachate. E, Mn/Sr. ratios from the U leachate. F, U_EF_ from the U leachate; *x*‐axis in log scale. G, Ce/Ce* ratios from the REE leachate. Vertical line of Ce/Ce* = 0.9 indicates the cutoff value for a locally oxic vs. anoxic environment, where Ce/Ce* < 0.9 suggests a locally oxic environment. H, Y/Ho ratios from the REE leachate. Vertical line of Y/Ho = 36 indicates the cutoff value for a primary seawater signal, where Y/Ho < 36 typically suggests detrital influence (Tostevin et al. 2016), although low Y/Ho values can also be characteristic of anoxic marine carbonates that are not detritally contaminated (Wallace et al. 2017). The gray band represents the BACE interval. N denotes the BACE and Z denotes a later positive δ^13^C_carb_ excursion defined in the original publications. Zircon U–Pb ages from Marusin et al. (2025).

Some 65 km to the south along the Kotuj River, our newly sampled Staraya Rechka and Manykai succession accumulated in a significantly more evaporitic setting (Marusin et al. 2025). As in the north, the Staraya Rechka Formation lies atop an angular unconformity separating it from the Mesoproterozoic Yusmastakh Formation. Notably, the Staraya Rechka Formation at this locality is over 3× thicker (~140 m) than in the northern exposure and is characterized by interbedded carbonate, evaporite, and shale. It is subdivided into three members, including the Kharyyalakh, Chimuka, and Kochokon members. The lower Kharyyalakh Member (~17 m thick) contains stromatolitic dolostone and laminated rippled dolosiltite and dololutite with minor evaporite levels (Figures 3 and 4, Figure S1). The middle Chimuka Member (~73 m thick) contains three thick intervals (up to ~10 m thick) dominated by gypsum, with four intervals of interbedded green shale and shaley dololutite, rippled dolosiltite, stromatolitic dolostone, and minor quartz sandstone. The upper Kochokon Member (~50 m thick) contains mostly evaporite‐free dolostone with hummocky and microbial bedding. The Staraya Rechka Formation in the southern exposure is interpreted to represent a shallow marine peritidal environment, supported by abundant evidence for episodic desiccation and development of ubiquitous sulfate evaporites, especially in the middle Chimuka Member. A disconformity of unknown duration separates the Staraya Rechka Formation from the overlying Manykai Formation, which is thinner (~65 m), less continuous, and preserves more evidence of evaporitic conditions than in the succession to the north, including several thick intervals of collapse breccia. Above the disconformity, ~1 m of conglomerate is presently overlain by ~10 m of shale and siltstones and ~20 m of covered outcrop (likely shale) corresponding to the lower parasequence (Beds I and II) in the northern succession. Above this there is ~40 m of dolostone and calcareous dolostone likely recording the development of a subtidal carbonate platform during sea‐level transgression (Kaufman et al. 1996). Mudstone and microbialite facies are associated with both synaeresis and desiccation cracks, gypsum pseudomorphs, and replacement nodules all indicative of elevated evaporation rates and variably hypersaline seawater (Figure S2). More open marine conditions are envisioned for the grainstone and stromatolitic facies.

#### 2.1.1 Paleontology and Chemostratigraphy

Small shelly fossils (SSFs) of Fortunian (and potentially Cambrian Stage II) aspect are abundant in the Manykai and Medvezhya formations (but not reported from the evaporitic Staraya Rechka Formation) of the well‐studied hypostratotype, including the sequential appearance of 86 discrete taxa of the *Anabarites trisulcatus*, *Purella antiqua*, and *Nochoroicyathus sunnaginicus* zones (Rozanov 1984; Rozanov and Zhuravlev 1992; Khomentovsky and Karlova 1993). While as many as 65 of these taxa also occur in broadly equivalent sections along the Aldan River of southeastern Siberia, biostratigraphic correlations between the two regions have been controversial because numerous species that first appear at a karstic unconformity in southeastern Siberia have first appearances over 30 meters of section in the Anabar Uplift. Carbon isotope variations in the northern Anabar Uplift succession potentially preserve the negative BACE event in the Staraya Rechka Formation (although see Marusin et al. 2025 for an alternative view) followed by an oscillating trend to increasingly ^13^C‐enriched carbonates of the Manykai and Medvezhya formations (Knoll et al. 1995; Kaufman et al. 1996) ending with a peak value around +6‰ (indicated as I′) near the Cambrian Stage II boundary (Figure 3D). Comparison of δ^13^C trends between the Anabar and Aldan sections demonstrates that 30 m of strata are missing at the karstic unconformity in the southeastern Siberia locality once considered as the stratotype for our understanding of the Cambrian Explosion of animals. These data illustrate that regional first appearances of SSFs in the Aldan region were controlled by the dynamics of regional sedimentation rather than a record of an evolutionary burst (Knoll et al. 1995; Kaufman et al. 1996).

Like the hypostratotype, the Staraya Rechka Formation along the Kotuj River preserves no known record of SSFs, although the calcareous algae *Renalcis* (Figure S1) was observed immediately above the Chimuka/Kochokon boundary. While there is ample evidence for bioturbation of Manykai sedimentary rocks in our southern Anabar Uplift exposure, including ichnofossils *Trepichthnus*, *Palaeophycus*, *Skolithos*, *Olenichnus*, and *Thalassinoides* (Figure S3), a comparable SSF record from the succession along the Kotuj River is not presently available. Algal organic sheets and body fossils of uncertain origin do appear here, including potential archeocyathids (that would indicate a Cambrian Stage II age; see Figure S3) in reef facies near the uppermost exposed beds. Given the presence of thick collapse breccia intervals at this locality, there may be considerable time hidden in cryptic unconformities within the evaporitic Staraya Rechka and basal Manykai formations at this locality, which complicates the use of high‐resolution carbon isotope trends to correlate these strata regionally and globally (e.g., Maloof et al. 2010).

**FIGURE 5:**
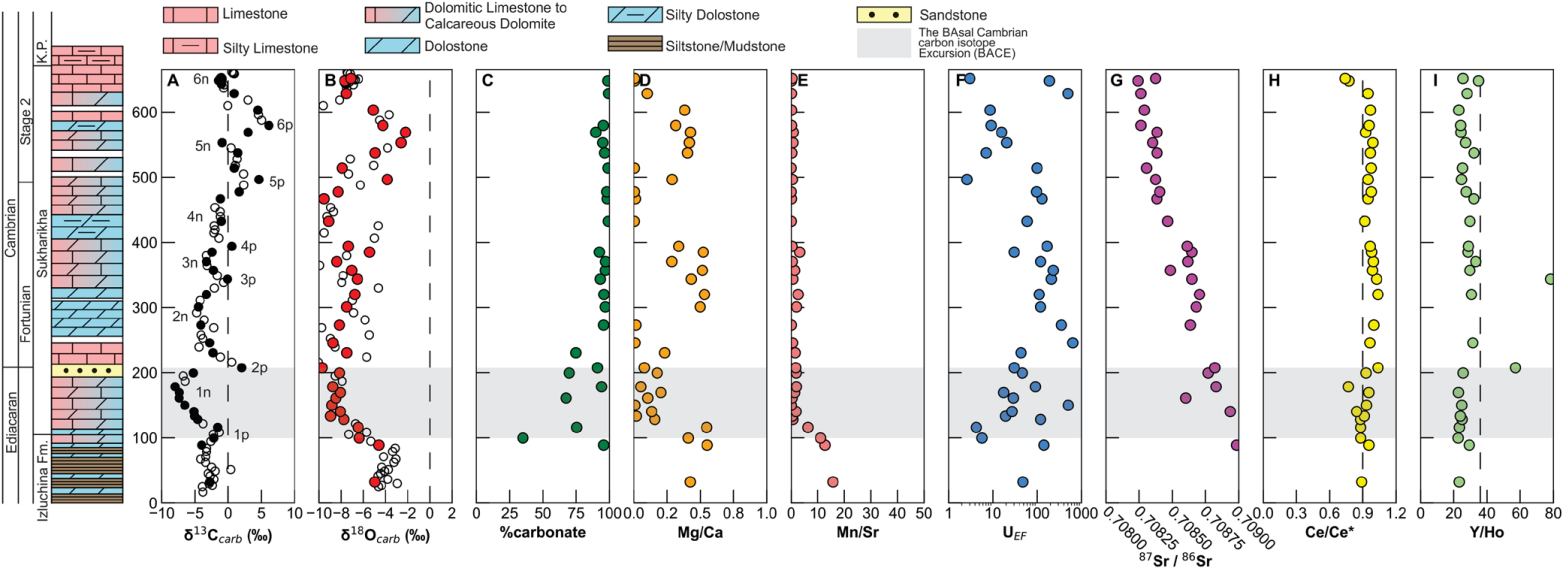
Stratigraphy and chemostratigraphy of the Izluchina and Sukharikha formations in the Sukharikha River section. A–B, δ^13^C_carb_ (A) and δ^18^O_carb_ (B) chemostratigraphy. δ^13^C_carb_ and δ^18^O_carb_ are referenced to V‐PDB. Closed circles represent samples analyzed for δ^238^U and elemental concentrations in this study. Open circles represent samples that were previously measured by Kouchinsky et al. (2007). C, Percentage of carbonate in the samples. D, Mg/Ca ratios from the U leachate. E, Mn/Sr ratios from the U leachate. F, U_EF_ from the U leachate; *x*‐axis in log scale. G, ^87^Sr/^86^Sr ratios. H, Ce/Ce* ratios from the REE leachate. Vertical line of Ce/Ce* = 0.9 indicates the cutoff value for a locally oxic vs. anoxic environment, where Ce/Ce* < 0.9 suggests a locally oxic environment. I, Y/Ho ratios from the REE leachate. Vertical line of Y/Ho = 36 indicates the cutoff value for a primary seawater signal, where Y/Ho < 36 typically suggests detrital influence (Tostevin et al. 2016), although low Y/Ho values can also be characteristic of anoxic marine carbonates that are not detritally contaminated (Wallace et al. 2017). K.P. = Krasny Porog Formation. The gray band represents the BACE interval. 1n refers to the BACE and the subsequent numbered δ^13^C_carb_ excursions were defined by Kouchinsky et al. (2007).

The carbon and oxygen isotope stratigraphy of the Kotuj River succession provides a more detailed window of variations in the much thicker dolostones of the Staraya Rechka Formation relative to the hypostratotype (Marusin et al. 2025), but the thinner and discontinuous record of Manykai carbonate δ^13^C oscillations in the southern Anabar Uplift is confusing (Figure 4A,B). In the Staraya Rechka Formation, dolomite δ^13^C values (Figure 4A) reveal a progressive increase from −2‰ to +2‰ in the first five meters of the succession prior to an unsampled recrystallized and covered interval. Above this level, δ^13^C values fall to values ranging between 0‰ and −2‰ upsection to the top of the Kharyyalakh Member. In the overlying and highly evaporitic Chimuka Member, carbonate carbon isotope values drop to a nadir of −6.5‰ at around 45 m before rising to between −4‰ and 0‰ between 65 and 80 m and continuing at this plateau throughout the ~50 m of the overlying Kochokon Member. Overall, we consider this negative δ^13^C excursion to be associated with the N1 or BACE event (Kaufman et al. 1996; Kouchinsky et al. 2007; contra Marusin et al. 2025; see Section 2.1.2). Oxygen isotope compositions of Staraya Rechka dolomites are notably enriched in ^18^O with δ^18^O values ranging between +1 and −2‰ with the heaviest values notably associated with the evaporite‐dominated Chimuka Member (Figure 4B). Oxygen isotope compositions of dolomites immediately below the disconformity separating the Staraya Rechka and Manykai formations drop to values near −4‰, and in the overlying limestones of the Manykai Formation, we observe further ^18^O depletion upsection with δ^18^O values ranging between −6‰ and −8‰. Immediately above the disconformity, Manykai conglomerates have δ^13^C compositions like those of underlying Staraya Rechka dolomites, but then there is a sudden drop in δ^13^C values to around −5‰ in overlying argillaceous limestones (Marusin et al. 2025). These authors did not report analyses of the discontinuous Manykai Formation above this level, so we present their carbon and oxygen isotope measurements in this study. Several covered intervals (some containing blocks of collapse breccia) were left unsampled, but in a relatively continuous package between ~155 and 180 m there is a general decline from values as high as +2‰ to a nadir of −6‰ ending at a stromatolitic reef complex containing the possible archeocyathid remains. Then above an 11‐m covered interval, two samples from the uppermost Manykai stromatolitic reef record δ^13^C values around +2‰ (Figure 4). While it is possible that the progressive negative δ^13^C trend between 155 and 180 m in the Kotuj River section reflects the negative excursion seen in Member III of the Manykai Formation in the hypostratotype (also see the 2p to 2n trend in the Sukharikha Formation of the Igarka Uplift; Kouchinsky et al. 2007; Figures 2 and 5), the lack of characteristic Fortunian δ^13^C oscillations in the Manykai Formation along the Kotuj River (potentially due to erosional truncation of the top of the unit) makes correlations highly speculative.

#### 2.1.2 Age Constraints

Recent detrital zircon U–Pb ages of sandstone beds within the Manykai and Staraya Rechka formations in the Kotuj River succession of the Anabar Uplift add temporal complexity to the negative carbon isotope anomaly considered here as the BACE event. The youngest population of detrital zircons from a sandstone at the base of the Manykai Formation immediately above the disconformity with the Staraya Rechka Formation yielded an age of 537 ± 3 Ma (Marusin et al. 2025), which is consistent with U–Pb detrital zircon age constraints (ca. 533–539 Ma) for the BACE in Laurentia. However, similar measurement of the youngest detrital zircons in a sandstone from the upper Staraya Rechka Formation in an eastern Anabar Uplift drillcore (Priyatkina et al. 2017) and from the uppermost Kochokon Member in the Kotuj River section (Marusin et al. 2025) yielded MDAs of ca. 543 ± 23 Ma (*n* = 3) and 547 ± 6 Ma (*n* = 7), respectively. Insofar as both ages are significantly older than the pre‐BACE detrital zircon U–Pb age constraint of 539.40 ± 0.23 Ma (*n* = 6 specifically picked from a suite of 140 detrital zircon grains measured by laser ablation techniques and subsequently analyzed by CA‐ID‐TIMS techniques) from Mexico (Hodgin et al. 2020), the negative carbon isotope anomaly in the Anabar Uplift has recently been considered as older than the Ediacaran–Cambrian transition section (Marusin et al. 2025). These authors suggest that the negative δ^13^C anomaly in the Staraya Rechka Formation might be related to older biogeochemical events in terminal Ediacaran time (Grotzinger et al. 2011; Cui et al. 2016; Bowyer et al. 2022).

In this study, we accept the long‐standing interpretation of the Staraya Rechka negative carbon isotope anomaly and its equivalents in Siberia as preserving the BACE event (see also Bowyer et al. 2023 whose correlations support this view) and assume that the time missing in the disconformity between the Staraya Rechka and Manykai formations is of short geological duration. In this view, transgression of the sea over the exposed tilted beds of the Mesoproterozoic Yustmastakh Formation led to the accumulation of carbonates and evaporites in shallow peritidal environments of the Staraya Rechka Formation in the Anabar Uplift and then further flooding of the marginal marine environment resulted in the deposition of more open marine facies of the Manykai Formation. Since underlying Ediacaran strata were either eroded or not deposited in this setting, there is no fossil evidence for the Ediacara biota, but the progressive appearance of SSFs in the northern Anabar Uplift and equivalents and of ichnotaxa of animals that deeply bioturbated the sediments pin the Manykai Formation to the early Cambrian Period. A critical observation of the Ediacaran stratigraphic record is that thick‐bedded evaporite deposits characterize the BACE interval throughout the Middle East, Pakistan, India, and Siberia and there is little to no evidence for bedded evaporite deposits throughout the rest of the Ediacaran Period. Supporting the view that the Staraya Rechka Formation preserves the BACE, the sulfur isotope composition of the thickly bedded evaporites in the Middle East and Asia from this interval reveal significant ^34^S enrichments (> 35‰; Fike and Grotzinger 2008; Meng et al. 2021; see also Holser 1977 that described the anomaly as the “Yudomski” event following sulfur isotope studies of Siberian evaporites), which are consistent with our sulfur isotope analyses of four evaporite samples from the Kharyyalakh and Chimuka members from the Kotuj River section (see Results and Table S1). In lieu of known older Ediacaran bedded evaporites and the consistency of the highly positive δ^34^S compositions from evaporite‐rich strata around the Ediacaran–Cambrian boundary, we accept the Staraya Rechka Formation in the Anabar Uplift as preserving the BACE event, although we recognize that uncertainties remain.

### 2.2 Geological Setting: Igarka Uplift

The sampled outcrop along the Sukharikha River in the Igarka Uplift, some 750 to 800 km to the southeast of our sections in the Anabar Uplift, contains the uppermost Izluchina, Sukharikha, and Krasny Porog formations (Figure 2). The Izluchina Formation is an 800‐ to 850‐m‐thick sedimentary succession generally composed of red‐colored planar‐laminated siltstones and mudstones with interbeds of planar‐laminated and cross‐bedded sandstones. The studied section contains the uppermost interval (83.5 m) where dolostone interbeds (Figure S4) become more abundant. The Izluchina Formation is interpreted to have accumulated in terrestrial alluvial and shallow marine deltaic environments (Kochnev et al. 2022). The transition between the Izluchina and Sukharikha formations represents a shift from terrestrial fluvial to shallow‐marine carbonate sedimentation during transgression (Rowland et al. 1998; Kochnev et al. 2022). The Sukharikha Formation in the studied section is 565 m thick and comprised of mixed limestone and dolostone. Thin interbeds and lenses of reddish‐gray siltstones and fine‐grained sandstones occur in the lowermost 20 m, while the rest of the formation is monotonous in composition and lacks conspicuous unconformity surfaces. In the lower part (124 m above the base of the Sukharikha Formation), there is a 20‐m‐thick interval composed of interbedded coarse‐ to medium‐grained sandstones with dolomitic cement, trough cross‐bedding, and wave‐rippled to planar‐laminated sandy dolostones. Hummocky cross‐stratified dolostones and dolomitic limestone, ranging from 1 to 9 m thick and separated by intervals of planar‐bedded limestones and planar‐laminated silty limestones are abundant in the interval between 200 and 240 m. In contrast, desiccation cracks indicative of exposure are common in the interval between 450 and 520 m. The contact with the overlying Krasny Porog Formation is a 1.5‐m‐thick gradual transition consisting of gray limestones like underlying strata of the Sukharikha Formation to pinkish‐gray and red parallel‐laminated silty limestones of the basal Krasny Porog Formation. The transition between the Sukharikha and Krasny Porog formations represents a transgressive flooding surface over shallow marine conditions to open shelf carbonate environments (Rowland et al. 1998; Kouchinsky et al. 2007). The Krasny Porog Formation (up to 150 m thick) is mostly composed of planar‐laminated and intensely bioturbated red silty limestones and marlstones.

#### 2.2.1 Chemostratigraphy and Paleontology

The Igarka succession has one of the best preserved and most complete records of Fortunian carbonate carbon isotope oscillations (Kouchinsky et al. 2007) including the negative BACE event followed upsection by six additional valleys and seven peaks that define an overall rising δ^13^C trend that extends into Cambrian Stage II (Figure 5). While from a chemostratigraphic perspective this succession is a Fortunian archetype (Maloof et al. 2010), its paleontological archive is extremely poor relative to that of the Anabar Uplift and elsewhere on the Siberian Craton (Astashkin et al. 1991; Luchinina et al. 1997; Rowland et al. 1998; Korovnikov et al. 2019; Marusin et al. 2023). Simple, but poorly preserved tubes of *Cambrotubulus*, an element of the earliest Fortunian *Anabarites trisulcatus* biozone (Peng et al. 2020), are found in the upper Izluchina Formation (Marusin et al. 2023), whereas the first appearance of a tri‐radial *Anabarites* comes from 240 m above the base of the Sukharikha Formation (Rowland et al. 1998). It is only in the uppermost 1.5 m of the Sukharikha Formation that a diverse SSF fauna of Cambrian Stage II aspect (*Nochoroicyathus sunnaginicus* biozone, including archeocyathids) suddenly occurs (Rowland et al. 1998), similar to observations along the Aldan River described above.

The ichnofossil record of the Sukharika Formation successions is equally depauperate. Deep‐tier vertical burrows of *Skolithos* and mid‐tier U‐shaped *Arenicolites* occur in the middle Sukharikha Formation (~284 m above the base; Korovnikov et al. 2019) at the studied section, but in the equivalent Plakhinskii section, *Thalassinoides* and *Olenichnus* have been described (Marusin et al. 2022), and from the Kulyumbe River section, *Heimdallia, Palaeophycus*, and *Thalassinoides* traces are reported. Otherwise, in the studied section only isolated calcimicrobe bioherms are noted in the upper 150 m of the Sukharikha Formation (Rowland et al. 1998).

## 3 Redox Systematics

### 3.1 Cerium Anomalies

To investigate local redox conditions, the use of rare Earth element (REE) abundances in carbonates has been successfully employed in many recent deep time studies. Among the REEs, cerium (Ce) is unique because it can exist in both trivalent and tetravalent oxidation states. Under oxic conditions, Ce(III) is partially oxidized to Ce(IV) and absorbed onto the surface of Mn oxides, where it no longer participates in solid solution exchange reactions, thereby depleting residual seawater in Ce relative to the other REEs (Tostevin et al. 2016). In this study, cerium anomalies (Ce/Ce*) are calculated by comparing the Post‐Archean Australian Shale (PAAS)‐normalized concentrations of Ce to its neighboring REEs using the following equation (Lawrence et al. 2006):

(1) $$\;\frac{\mathrm{Ce}}{\mathrm{Ce}}*=\frac{{\left[\mathrm{Ce}\right]}_{\text{PAAS}}}{\left(\frac{{\left[\mathrm{Pr}\right]}_{\text{PAAS}}^{2}}{{\left[\mathrm{Nd}\right]}_{\text{PAAS}}}\right)}$$

Negative Ce anomalies are ubiquitous in the modern, well‐oxygenated ocean, although the magnitude of the anomaly varies within and between ocean basins (De Baar et al. 1985; De Baar 1991) and can respond to changes in water column redox on a meter scale (De Carlo and Green 2002). Under anoxic conditions, Ce remains in a trivalent state and is not removed from the water column, which results in a Ce/Ce* ratio that is greater than or equal to 0.9 (Tostevin et al. 2016). However, positive Ce anomalies can be generated under manganous conditions in which the tetravalent Ce contacts an anoxic and manganous fluid (potentially associated with a redoxcline) and is reduced to the trivalent state. Additionally, the preferential stabilization and formation of carbonate‐Ce(IV)‐complexes under alkaline conditions may also bring about a positive Ce/Ce* (Möller and Bau 1993; Pourret et al. 2008). Another powerful REE proxy is the yttrium to holmium (Y/Ho) ratio, which can be used to validate a primary seawater signal within carbonates as Ho is scavenged rapidly from seawater relative to Y due to differences in surface complexation. Thus, a higher Y/Ho (> 36) is used to indicate a primary seawater signal (Liu and Byrne 1995; Nozaki et al. 1997; Nothdurft et al. 2004; Tostevin et al. 2016).

### 3.2 Uranium Isotopes

The two most abundant long‐lived isotopes of uranium are ^235^U and ^238^U, and uranium occurs in nature predominantly in two oxidation states, U(IV) and U(VI). U(IV) is highly insoluble and particle‐reactive under anoxic conditions, whereas U(VI) is soluble and remains mobile in oxygenated environments. Uranium has a residence time of ~400–500 kyr in the modern ocean (Ku et al. 1977), and due to its long residence time compared to the ocean mixing time, it is well‐mixed throughout the global ocean resulting in a consistent open ocean concentration of ~3.2 ng/g and a δ^238^U value of −0.379‰ ± 0.023‰ (Owens et al. 2011; Kipp and Tissot 2022). The primary source of U to the oceans is oxidative weathering of continental rocks. Uranium has a variety of sinks in the ocean including oxic, suboxic, anoxic, euxinic, and carbonate sediments, as well as both high‐ and low‐temperature hydrothermal systems and basalt alteration (Tissot and Dauphas 2015; Lau et al. 2019). Anoxic and euxinic sediments impart the largest degree of δ^238^U fractionation by preferentially removing ^238^U from seawater through reduction of U(VI) to insoluble U(IV) (Andersen et al. 2014; Holmden et al. 2015; Noordmann et al. 2015; Rolison et al. 2017), with fractionation resulting from nuclear volume effects. As a result, the more extensive the area of seafloor covered by anoxic and/or euxinic sediments, the greater the removal of the heavy ^238^U isotope, resulting in the enrichment of the light ^235^U isotope and lower seawater δ^238^U compositions (Lau et al. 2019; Zhang et al. 2020). Thus, periods of widespread ocean anoxia are characterized by lower seawater δ^238^U values, whereas periods of well‐oxygenated oceans are characterized by higher seawater δ^238^U values like those of the upper continental crust and riverine inputs. Highly productive and/or euxinic environments impart the largest degree of uranium isotope fractionation in the modern ocean (Holmden et al. 2015; Noordmann et al. 2015; Rolison et al. 2017; Bruggmann et al. 2022), such that uranium isotopes are likely best used specifically as a proxy for the seafloor extent of highly productive and/or euxinic environments in the geologic past, instead of just generally anoxic environments (Rutledge et al. 2024; Kunert et al. 2025).

Well‐preserved carbonate sediments can be a reliable archive for the δ^238^U composition of the seawater from which they form. In most cases, modern biogenic precipitates in the Bahamas carbonate platform incorporate uranium with little to no isotopic fractionation (~0.09‰; Romaniello et al. 2013; Chen et al. 2018). However, analysis of shallow bank top syndepositional carbonate sediments reveals a positive offset of 0.26‰ ± 0.10‰ in δ^238^U relative to seawater (Chen et al. 2018; Tissot et al. 2018). This diagenetic offset exists due to the authigenic enrichment of isotopically heavy U(IV) in sediments in sulfidic pore waters, which are prevalent in shallow bank top sediments due to the degradation of organic matter. Post‐depositional processes such as burial diagenesis and mineralogical transformations from aragonite to calcite and early diagenetic dolomite often result in minimal δ^238^U alteration in marine carbonates due to the low solubility of the element under reducing diagenetic conditions (Chen et al. 2018; Tissot et al. 2018), although burial dolomite can be substantially offset from original seawater values (Hood et al. 2018). Thus, post‐depositional marine carbonates can retain their syndepositional δ^238^U signatures (Chen et al. 2018). A recent study of modern and Neogene carbonates from the South China Sea suggested a similar offset between carbonates and seawater of 0.24‰ ± 0.24‰, although different modes of diagenesis produced slightly different magnitudes of fractionation (Xiong et al. 2025). Importantly, data from both the Bahamas and South China Sea show no statistically significant difference in δ^238^U values between calcite and dolomite, implying that both limestones and dolostones can faithfully record the primary or early diagenetic δ^238^U signature (Chen et al. 2018; Tissot et al. 2018; Xiong et al. 2025).

## 4 Methods

### 4.1 Sampling

For this integrated redox study of the BACE and Fortunian Stage of the Cambrian Period, archived stratigraphic samples of the Staraya Rechka, Manykai, and Medvezhya formations collected from the northern Anabar Uplift along the Kotujkan River in 1992 by A. Knoll, J. Grotzinger, and M. Semikhatov were selected for elemental, organic carbon, and pyrite sulfur isotope analysis. New samples from the southern Anabar Uplift were collected in 2021 by V. Marusin and N. Bykova from the Staraya Rechka and Manykai formations along the Kotuj River, and in 2018, samples were collected by V. Marusin and B. Kochnev from the Izluchina, Sukharikha, and Krasny Porog formations along the Sukharikha River in the Igarka Uplift. In all sample suites, weathered surfaces were removed and any obvious diagenetic features such as veins were avoided before samples were crushed into a fine powder in an agate ball mill crushing jar. Carbonates from the southern Anabar and Igarka uplifts were analyzed for their δ^13^C and δ^18^O compositions at the Analytical Center for Multielemental and Isotope Research in Novosibirsk and are reported in Marusin et al. (2023, 2025).

### 4.2 Analytical Techniques

#### 4.2.1 Organic Carbon and Pyrite Sulfur Isotopes

Powders of bulk carbonate samples were repeatedly acidified with 3M HCl to remove carbonate, washed with ultra‐pure Milli‐Q (18.2 MΩ) water, and solutions decanted to concentrate organic matter and pyrite in residues. The residues were dried at 80°C overnight and then quantified gravimetrically to determine wt.% carbonate and wt.% residue in the bulk powders. Organic carbon abundance and isotopic composition of residues were determined by combustion of ~1–3 mg of homogenized residue packed and folded into small tin cups. The organic carbon was converted to CO_2_ by combustion with a pulse of O_2_ in a Eurovector EA3000 at 1040°C through a column packed with silvered cobaltous cobaltic oxide and chromium oxide. The CO_2_ analyte was carried in a ~75 mL/min stream of UHP He through a reduction column packed with elemental copper wires at 650°C to reduce trace NO_x_ to N_2_, a magnesium perchlorate water trap, and a GC column at 50°C before entering the source of the Elementar Isoprime continuous flow isotope ratio mass spectrometer (IRMS). The method included the measurement of m/z 44, 45, and 46 for a CO_2_ reference gas (RT 20–50 s) and the CO_2_ analyte (RT ~210–350 s). Carbon isotope ratios were determined by comparing integrated peak areas of m/z 44, 45, and 46 for the reference and sample CO_2_ pulses, relative to the baseline that is approximately 2 × 10^−10^ A. Offset corrections and uncertainty for carbon abundance (< 1%) and isotope (< 0.2‰) composition in each analytical session were determined by the average and standard deviation, respectively, of five urea analyses at the beginning of each day to test for system suitability, along with two urea measurements between batches of 10 unknown samples. Isotopic results are expressed in the delta notation as per mil (‰) deviations from the Vienna Pee Dee Belemnite (V‐PDB) standard. Total organic carbon (TOC) in pre‐acidified bulk powders was determined by multiplying the wt.% C in the residue by the wt.% residue.

Sulfur isotope abundances of residues measured by combustion of ~5–10 g (accurately weighed and placed into tin cups with ~0.5 mg of V_2_O_5_ added as an additional oxidant) to SO_2_ with the same Eurovector EA3000 in line with an Elementar Isoprime IRMS. The folded tin cups were sequentially dropped with a pulsed O_2_ purge of 12 mL into a catalytic combustion furnace operating at 1030°C. The frosted quartz reaction tube was packed with elemental copper wire for quantitative oxidation and O_2_ resorption. Water was removed from the combustion products with a 10‐cm Mg(ClO_4_)_2_ trap, and the SO_2_ was separated from CO_2_ with a 0.8‐m PTFE GC column packed with Porapak 50–80 mesh heated to 115°C. The effluent from the elemental analyzer (EA) was introduced in a ~75 mL/min stream of UHP He to an Elementar Isoprime IRMS through a SGE splitter valve. Timed pulses of SO_2_ reference gas (Air Products 99.9% purity, ∼3 nA) were introduced at the beginning of the run using an injector connected to the IRMS. The cycle time for these analyses was 210 s with reference gas injection as a 30‐s pulse beginning at 20 s. Sample SO_2_ pulses begin at 110 s and return to baseline values between 150 and 180 s, depending on sample size and column conditions. Sulfur isotope ratios were determined by comparing integrated peak areas of m/z 66 and 64 for the reference and sample SO_2_ pulses, relative to the baseline that is approximately 1 × 10^−11^ A. Isotopic results are expressed in the delta notation as per mil (‰) deviations from the Vienna Canyon Diablo Troilite (V‐CDT) standard. Two NBS 127 barite standards and two IAEA NZ1 silver sulfide standards were measured between each set of 10 samples for drift and offset corrections; uncertainties for each analytical session based on these standard analyses were determined to be better than 0.3‰. Total sulfur (TS) in pre‐acidified bulk powders was determined by multiplying the wt.% S in the residue by the wt.% residue.

#### 4.2.2 Major, Trace, and Rare Earth Elements

To determine the major, minor, and trace element concentrations of the new samples from the Anabar and Igarka uplifts, solutions resulting from the uranium acidifications (see Section 4.2.4) were used. A 200 μL aliquot of each solution was taken and added to individual 15 mL centrifuge tubes, which were diluted up to 10 mL with 2% trace‐metal grade (TMG) HNO_3_. Major, minor, and trace elements were analyzed on the Thermo Scientific Quadrupole iCAP‐Q ICP‐MS using both standard and kinetic energy dispersion (KED) modes and He in the collision cell. A custom internal standard composed of 400 ppb Sc and 200 ppb of Ge, In, and Bi was introduced with the sample at equal flow rates to the spray chamber to account for sample matrix variation and the high‐frequency ionization physics inherent to plasma techniques. External calibration relies on in‐house calibration solutions prepared gravimetrically from certified single‐element standards, and the calibration is validated with several QA/QC solutions of known composition. For quality control, samples were run alongside several in‐house standards. Reported uncertainties for the analytical suite are better than 6% RSD.

The REE procedure used follows the protocol detailed in Tostevin et al. (2016). Approximately 25 mg of sample powder was weighed and placed in fresh 2 mL disposable centrifuge tubes. The samples were rinsed with 18.2 MΩ Milli‐Q water at 25°C, agitated for 30 min, centrifuged at 5000 rpm for 10 min, and the water discarded. Next, 312.5 μL of 2% TMG HNO_3_ was added to each sample, which was then agitated and left to react overnight. The following morning, samples were centrifuged at 5000 rpm for 10 min, the supernatant was discarded, and the process was repeated with 625 μL of 2% TMG HNO_3_. The supernatant from each sample was decanted into fresh 15 mL centrifuge tubes and diluted up to 10 mL with 2% TMG HNO_3_. Rare Earth elements in these solutions were similarly measured on a Thermo Scientific iCAP‐Q ICP‐MS and quality control was checked using several in‐house standards. Reported uncertainties for the analytical suite are better than 6% RSD.

#### 4.2.3 Strontium Isotopes

For analysis of strontium isotopic (^87^Sr/^86^Sr) composition, only limestone/calcite samples were selected for extraction and measurement. Micro‐drilled powders (ca. 5–10 mg) were leached three times in 0.2 M ammonium acetate (pH = ∼8.2) to remove exchangeable Sr from non‐carbonate minerals and then rinsed three times with Milli‐Q water. The leached powder was centrifuged, decanted, and acidified with doubly‐distilled 0.5 M acetic acid overnight to remove strontium from the carbonate crystal structure. The supernatant was centrifuged to remove insoluble residues and then decanted, dried, and subsequently dissolved in 200 μL of 3 M HNO_3_ (doubly‐distilled acid was used here and for the remainder of the procedure). Strontium separation by cation exchange was carried out using a small polyethylene column containing ~1 cm of Eichrom Sr‐specific resin. The column was rinsed with 400 μL of 3 M HNO_3_ before the dissolved sample was loaded. After loading, the sample was sequentially eluted with 200 μL of 3 M HNO_3_, 600 μL of 7 M HNO_3_, and 100 μL of 3 M HNO_3_ to remove the Ca, Rb, and REE fractions. The Sr fraction was removed by elution with ∼800 μL of 0.05 M HNO_3_ and the resultant eluate collected and dried. The strontium was then dissolved in 2% HNO_3_ for isotopic analysis on a Thermo Scientific Neptune Plus multi collector‐inductively coupled plasma‐mass spectrometer (MC‐ICP‐MS). The purified Sr solutions were desolvated using a Cetac Aridus II with a nebulizer (uptake 50 μLmin^−1^). Sweep and nitrogen gas flow were optimized for each analytical session. The NBS987 strontium standard was analyzed repeatedly with each batch of samples, along with an in‐house Sr reference solution. Concentrations of standards were 50 ppb. Samples were diluted with 2% HNO_3_ to match the concentration of the standards, with a 50‐ppb solution typically yielding 4 V of total Sr signal. Masses at 84, 85, 86, 87, and 88 were measured with 10^11^ Ω resistors. Each individual measurement consisted of 40 cycles in one block with 4.194 s integration time for each cycle. Potential interferences from ^82^Kr and ^83^Kr on ^84^Sr and ^86^Sr were determined by the measurement of these masses while aspirating pure 2% HNO_3_. Instrumental mass fractionation was accounted for by the measurement of ^88^Sr/^86^Sr corrected to the true value of 8.3786. Measurement at mass 85 allowed evaluation of the potential interference of ^87^Rb on ^87^Sr. The average and standard deviation of ^87^Sr/^86^Sr in NBS 987 over three consecutive analytical sessions was 0.710243 ± 0.000011 (certified value = 0.71034 ± 0.00026).

#### 4.2.4 Uranium Isotopes

Samples were prepared for δ^238^U analysis following established laboratory procedures (Gilleaudeau et al. 2019; Cherry et al. 2022). Approximately 2 g of each sample was acidified overnight with 35 mL of 1 M HNO_3_ and 5 mL of concentrated HNO_3_ (all acids were TMG). The samples were centrifuged and the supernatant decanted into a separate centrifuge tube. Aliquots of the supernatant (~200 μL) were taken and diluted to 10 mL with 2% HNO_3_ with these solutions analyzed for major and trace element abundances using an iCAP‐Q ICP‐MS as described above. After acidification of the initial sample, the insoluble residue was rinsed with Milli‐Q water to remove residual acid, dried at 80°C, and gravimetrically quantified to calculate the wt.% carbonate. Sample specific amounts of the supernatant were then dried down in Teflon beakers (aiming for 300 ng U) before an appropriate amount of Institute for Reference Materials and Measurements (IRMM) ^233^U/^236^U double‐spike was added to each (0.8 mL of a 10.7 ppb double‐spike solution per 500 ng of U). The samples were sequentially digested with reverse aqua regia, H_2_O_2_, and concentrated HNO_3_ and then were partially dried down and redissolved in 3 M HNO_3_.

Uranium was separated through two rounds of ion exchange chromatography (Zhang et al. 2020). The first round utilized UTEVA (Uranium and TetraValents Actinides) resin, while the second round used DGA (Diglycolamine) resin to clean up any residual Na and Fe that persisted after the UTEVA column. Uranium isotope analysis was performed on a Thermo Scientific Neptune Plus MC‐ICP‐MS with an Aridus II inlet system. Certified Reference Material (CRM)‐145 and CRM‐129a were used during the analytical session, with the CRM‐145 standard analyzed after every two samples to allow for sample‐standard bracketing when calculating the sample δ^238^U values. CRM‐129a bracketed by two CRM‐145 standards was run after every 10 samples to ensure long‐term reproducibility. Our mean δ^238^U value for CRM‐129a was −0.75‰ ± 0.13‰ (2SD, *n* = 15), in excellent agreement with published values from multiple laboratories (Gilleaudeau et al. 2019; Cherry et al. 2022). Each sample was analyzed at least twice, and analytical precision is reported as 2SD of replicate measurements. The average analytical precision was ±0.09‰ and ±0.08‰ for samples from the Kotuj and Sukharikha River sections, respectively.

## 5 Results

### 5.1 Kotujkan River

The sedimentology, sequence architecture, biostratigraphic, and chemostratigraphic characteristics of the northern Anabar succession were previously reported (Knoll et al. 1995; Kaufman et al. 1996) including high‐resolution time‐series elemental (Mg/Ca and Mn/Sr) and carbon and oxygen isotope variations through the BACE and overlying Fortunian Stage. In this study, we provide new organic carbon and pyrite sulfur abundance and isotope composition data on the same sample suite. Acidification of powders allowed for the determination of wt.% carbonate (Table S1; Figure 3), which was > 95 wt.% in the Staraya Rechka samples and > 75 wt.% in those from the Manykai Formation. The wt.% TOC of samples was generally low (< 0.25 wt.%) with the Manykai samples having only slightly enriched abundances relative to those of the Staraya Rechka Formation. Organic carbon δ^13^C compositions in the succession range between −34‰ and −26‰ with slightly greater ^13^C depletion noted in the evaporitic Staraya Rechka interval relative to that in the more open marine Manykai facies (Figure 3). It is difficult to ascertain whether trends in carbonate and organic carbon isotope composition are clearly coupled given the narrow range of δ^13^C_org_ compositions, but this appears to be the case in the intervals between 0 and 10 m in the Staraya Rechka Formation, in Manykai strata in two intervals associated with member III (70–80 m) and members V/VI (110–120 m), and at the top of the measured succession in the Medvezhya Formation (155–165 m). Analyses of pyrite sulfur in residues reveal that although samples contain < 0.3 wt.% sulfur, there are profound temporal changes in δ^34^S compositions through the northern Anabar Uplift succession. At the base of the Staraya Rechka Formation, values start around −15‰ but then progressively rise over 40 m of evaporite‐rich strata to as high as +28‰ at the top of the formation. In the Manykai Formation, pyrite δ^34^S values reveal a remarkable 50‰ range of values depicting clear stratigraphic trends based on closely spaced sampling. The most ^34^S depleted value of −22‰ is noted in Member III, which is associated with the minor positive δ^13^C excursion labelled Z (Knoll et al. 1995; Kaufman et al. 1996), while a strong positive trend to a peak δ^34^S value of +30‰ is noted across the Member IV/V transition where there is a slight negative carbonate δ^13^C excursion. This sulfur isotope peak is followed by a deep valley in thrombolite facies of Member V to values as low as −20‰ at ~100 m in height after which values rise to as high as +25‰ before another sharp negative sulfur isotope excursion in Member IX (the Koril Member) again associated with thrombolite (or calcimicrobe) facies.

### 5.2 Kotuj River

In the Kotuj River section of the southern Anabar Uplift, Staraya Rechka dolostones in the Kharyyalakh Member are predominantly composed of broad stromatolitic mounds that are often pockmarked with vugs from pre‐existing evaporite minerals, whereas the overlying Chimuka Member is dominated by thick‐bedded and nodular evaporites with thin interbedded dolomites (Figure 4, Figure S1). The Kochokon Member predominantly contains dolomitic grainstones with silica and bitumen‐filled vugs, broad stromatolitic mounds, and breccias along with an increase in shale horizons towards the top of the member. Thin section observations of Staraya Rechka carbonates indicate that they mainly consist of pure dolomicrite or dolomicrospar with scattered micritic clots that are likely calcimicrobes (including *Renalcis*) or organic sheets that could be the remains of algae or microbial mats. The dolomicritic matrix is generally uniform, shows low detrital content, and preserves primary sedimentary fabrics, including intermixed evaporite minerals as laths, lozenges, or vugs that are preferentially leached away in surface exposures.

In contrast, bedded limestone and dolomitic limestone in the overlying Manykai Formation are often argillaceous, recrystallized, and bioturbated. While laminated and thinly‐bedded limestones dominate the lower siliciclastic‐rich interval, the middle reach of the Manykai Formation is characterized by cross‐bedded grainstones and three prominent stromatolitic reefs near the top of the exposed succession. Manykai grainstones have silica and bitumen‐filled vugs, and mudstones are typically vuggy due to the dissolution of pre‐existing evaporite minerals (Figure S2) like the same muddy facies in the underlying Staraya Rechka Formation. Bedding plane features in Manykai strata, including synaeresis and desiccation cracks, attest to the evaporitic nature of the succession in the southern Anabar Uplift (Figure S2). Manykai bedding surfaces also document a wide range of ichnofabrics (Figure S3) left behind by animals that bioturbated the sediments, which arguably facilitated the recrystallization of original micritic fabrics. Petrographic observations reveal that the coarser grained lithologies often contain peloids of various sizes floating in a sparry matrix along with disseminated hematite that is likely the oxidation product of pre‐existing pyrite.

**FIGURE 6:**
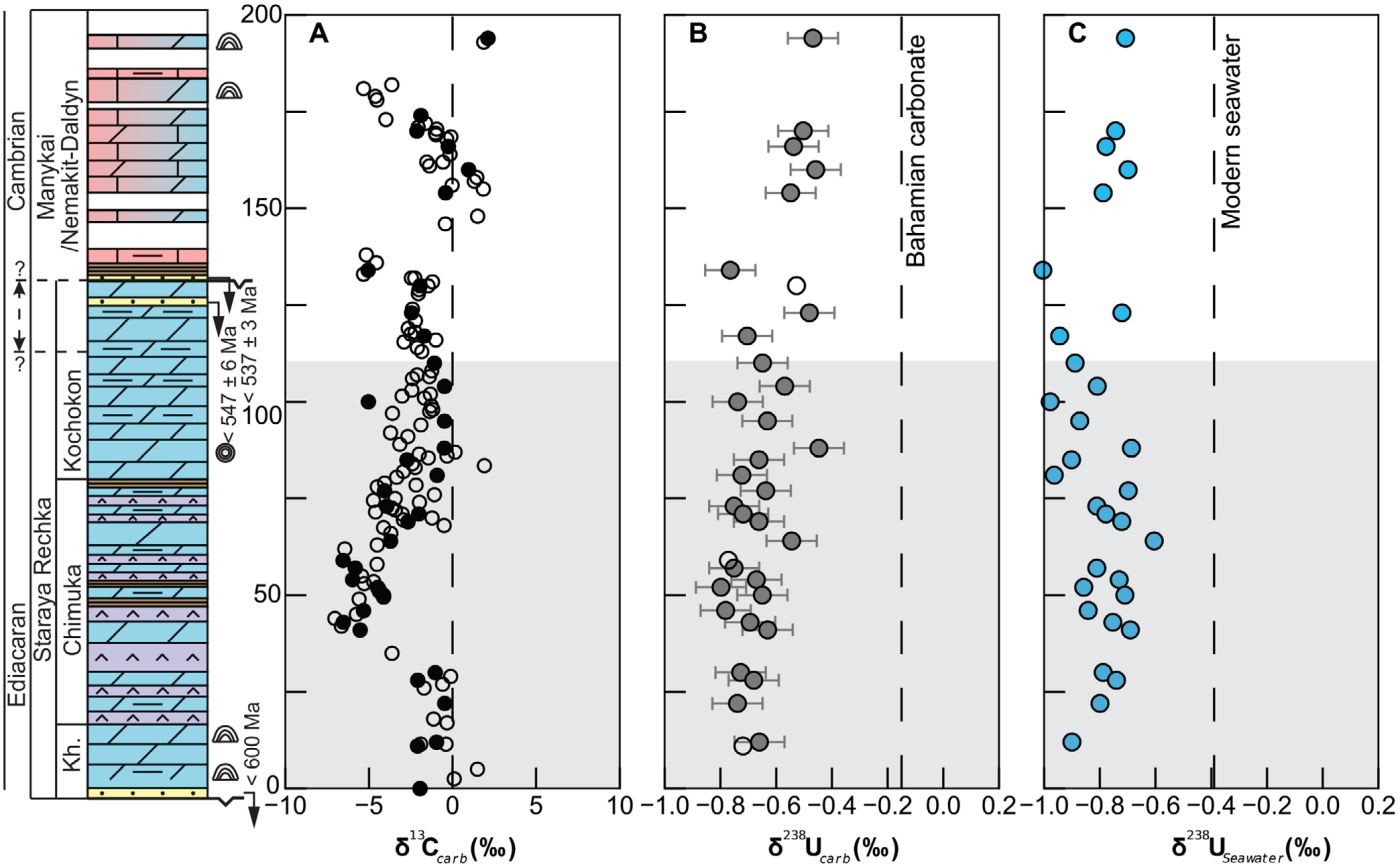
Stratigraphy, δ^13^C_carb_, δ^238^U_carb_, and reconstructed seawater δ^238^U from the Kotuj River section. (A) δ^13^C_carb_ from Figure 4A. (B) Carbonate δ^238^U values measured in this study. The dashed line represents the δ^238^U median of modern Bahamian carbonate (−0.15‰, Chen et al. 2018). Uncertainties (±0.09‰) are calculated from the average of duplicate analyses for the entire sample set in this section. The open circles indicate that the samples are rejected for reconstructing seawater δ^238^U because of likely contamination by isotopically heavy U(IV), diagnosed by Mo/(Ca + Mg) > 0.1 × 10^−6^. (C) Reconstructed seawater δ^238^U values, applying a 0.24‰ offset to all samples, except for a 0.06‰ offset in the Chimuka Member of Staraya Rechka Formation based on highly evaporative conditions in this formation (see main text for the details). Although applying a 0.24‰ offset between seawater and carbonates is effective when applied to large datasets of modern carbonates, we recognize that there is substantial variability among individual samples (Chen et al. 2018; Xiong et al. 2025). Thus, the sample‐by‐sample correction to seawater δ^238^U values presented here is meant to be interpreted broadly, with less emphasis placed on small inter‐sample variability. The dashed line represents the modern seawater δ^238^U value (−0.38‰, Kipp and Tissot 2022). The gray band represents the BACE interval. Zircon U–Pb ages from Marusin et al. (2025). For legend symbols, refer to Figure 4.

Geochemical results from the Kotuj River section of the Staraya Rechka and Manykai formations are tabulated and illustrated (see Table S1, Figure 4) in this study. The percentage of carbonate is similar across the entire section, with all samples (except one in the basal Manykai Formation) having > 85 wt.%. Magnesium/calcium ratios between 0.4 and 0.5 indicate pervasive dolomitization in the Staraya Rechka Formation, followed by a decline to near 0 in the overlying Manykai limestones. Manganese/strontium ratios are very low in samples above 50 m in the section, but below this level several of the Staraya Rechka samples have elevated values. The sulfur isotope composition of four gypsum samples from the Staraya Rechka Formation, including one from the lower Kharyyalakh Member and three from the middle Chimuka Member, range between +34.1‰ and +36.8‰ (Table S1). Uranium concentrations range from 0.09 to 2.14 ppm, and calculated U enrichment factors (EF) were determined to assess the degree of authigenic vs. detrital U in our samples. The EF was calculated as (U/Al)_sample_/(U/Al)_UCC_ where UCC refers to upper continental crust values (U = 2.8 ppm, Al = 8.15 wt.%; Rudnick and Gao 2003). In the Kotuj River section, U_EF_ ranges from 6 to 642, with a median value of 58. Carbonate δ^238^U values are uniformly low in the Kotuj River section, with a median value of −0.66‰ (Figure 6). For the Staraya Rechka Formation, the median δ^238^U value is −0.69‰, but we do note a slight increase in values across the interpreted BACE interval. For the Manykai Formation, the median δ^238^U value increases to −0.50‰ with no clear stratigraphic trend or outliers. In the Kotuj River section, Ce/Ce* values range from 0.99 to 1.50 and Y/Ho ratios range from 15.9 to 63.9.

### 5.3 Sukharikha River

In the Sukharikha River section, Izluchina, Sukharikha, and Krasny Porog carbonates preserve a wide range of sedimentary fabrics that provide clues to depositional environments. Microbialaminites and grainstones composed of intraclasts, peloids, grapestones, and oolites characterize the thin Izluchina dolostones near the top of the formation. Along with acicular precipitate fabrics (Figure S4) observed on weathered surfaces, these coated grains suggest elevated carbonate saturation states in a shallow and perhaps evaporitic marine setting. Thin section observation of several closely spaced Izluchina dolostones reveals possible evaporite crystals and irregular nodules displacing dolomicrite fabrics supporting a peritidal depositional environment with high rates of seawater evaporation. Sea level transgression over Izluchina strata resulted in more open marine grainstone, intraclastic, stromatolitic, and microbial facies preserved in mixed limestone and dolomitic limestone lithologies. Abundant desiccation cracks on exposed surfaces of limestones between 460 and 520 m suggest sea level regression through this interval of the Sukharikha Formation. The lithological transition from the Sukharikha to the Krasny Porog Formation is gradual and unremarkable, but the color change from grey to oxidized red is pronounced. Thin section observations indicate that lower Sukharikha Formation limestones are predominantly composed of micritic peloids and intraclasts with microsparitic cements and minor pyrite, whereas the dolomitic limestones higher in the succession preserve scattered dolomite rhombs (Figures S4 and S5). Micritic intraclasts are often cemented with euhedral dolospar in the interval with abundant desiccation cracks. The red silty limestones of the Krasny Porog Formation are richly fossiliferous packed with the recrystallized remains of SSFs as well as archeocyathids surrounded by the calcareous algae *Epiphyton*.

Geochemical measurements of the Sukharikha River succession provide an additional means to evaluate the extent of preservation of samples and their ability to serve as a proxy for environmental conditions through the Fortunian Stage. Most of the collected samples from the Sukharikha and Kransy Porog formations are rich in carbonate (> 70 wt.%) but a few from the Izluchina Formation are as low as 35 wt.%. Mirroring this observation, Mn/Sr ratios are less than 10 in all samples except for the lowermost three Izluchina samples in the section. Based on Mg/Ca ratios, the intervals of the most pervasive dolomitization occur in the Izluchina and Sukharikha intervals between 300–400 m and 500–600 m height in the measured section. The carbon isotope compositions of samples collected from the Sukharikha River locality for this study record the same oscillations in δ^13^C compositions through the Sukharikha Formation as reported earlier (Kouchinsky et al. 2007) albeit at a lower stratigraphic resolution (Figure 5). The first valley (1n) has a δ^13^C nadir of −8.0‰ and is interpreted to represent the BACE. Stratigraphically higher, there is an overall trend towards more positive δ^13^C values through the Fortunian, with each negative perturbation (2n–6n) decreasing in depth while the positive perturbations (2p–6p) progressively increase in magnitude upsection. We also present oxygen isotope abundances and notably, the Izluchina Formation dolostone samples below the BACE are ^18^O‐enriched, similar in magnitude to the dolomites of the Staraya Rechka Formation in both Anabar Uplift localities associated with highly evaporitic conditions. During the BACE in the Igarka Uplift, the oxygen isotopes dive down to as low as −9‰ and remain strongly depleted in ^18^O until near the top of the Fortunian Sukharikha Formation where values rise as high as −2‰, before falling again to −8‰ in the red Krasny Porog limestones of Cambrian Stage II. Across the Igarka succession, it appears that oxygen and carbon isotope compositions broadly covary. In contrast, ^87^Sr/^86^Sr compositions of samples through the Sukharikha and Krasny Porog formations reveal a systematic stratigraphic decrease through the Fortunian and into Cambrian Stage II with values falling from ~0.70900 to ~0.70825 over the course of the section.

**FIGURE 7:**
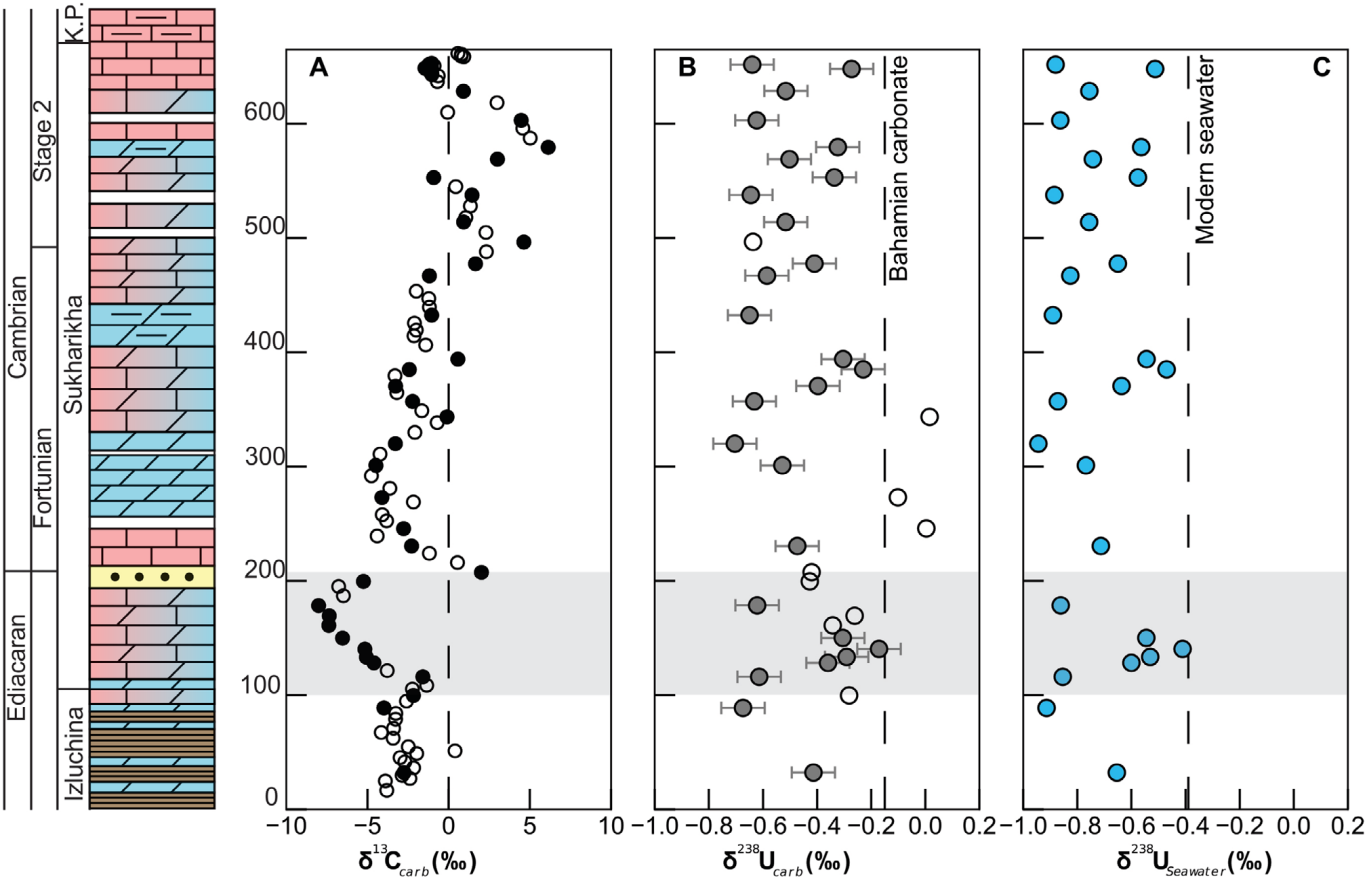
Stratigraphy, δ^13^C_carb_, δ^238^U_carb_, and reconstructed seawater δ^238^U from the Sukharikha River section. (A) δ^13^C_carb_ from Figure 5A. (B) Carbonate δ^238^U values measured in this study. The dashed line represents the δ^238^U median of modern Bahamian carbonate (−0.15‰, Chen et al. 2018). Uncertainties (±0.08‰) are calculated from the average of duplicate analyses for the entire sample set in this section. The open circles indicate that the samples are rejected for reconstructing seawater δ^238^U because of likely contamination by isotopically heavy U(IV), diagnosed by Mo/(Ca + Mg) > 0.1 × 10^−6^. (C) Reconstructed seawater δ^238^U values, applying a 0.24‰ offset to all samples (see main text for the details). Although applying a 0.24‰ offset between seawater and carbonates is effective when applied to large datasets of modern carbonates, we recognize that there is substantial variability among individual samples (Chen et al. 2018; Xiong et al. 2025). Thus, the sample‐by‐sample correction to seawater δ^238^U values presented here is meant to be interpreted broadly, with less emphasis placed on small inter‐sample variability. The dashed line represents the modern seawater δ^238^U value (−0.38‰, Kipp and Tissot 2022). K.P. = Krasny Porog Formation. The gray band represents the BACE interval. For legend symbols, refer to Figure 5.

The extent of local and global redox through the Fortunian Stage is evaluated in this study through the lens of Ce anomalies and uranium isotope compositions of these Siberian carbonates. Uranium concentrations range from 0.07 to 8.41 ppm, and using the calculation described above, the U_EF_ in the Sukharikha River section ranges from 3 to 506 with a median value of 54. The median δ^238^U value for the Sukharikha River section is −0.42‰ (Figure 7), with substantially more stratigraphic variation than is observed in the Kotuj River section (Figure 6). While more data is needed to confirm trends, the most ^238^U enriched intervals appear to be associated with the 1n, 3n, and 5n carbon cycle excursions, although none of these rise to the level measured in carbonates accumulating in the modern well‐oxygenated ocean. In the Sukharikha River section, Ce/Ce* values range from 0.74 to 1.39 and Y/Ho ratios range from 22.7 to 78.4 (Figure 5).

## 6 Discussion

### 6.1 Depositional and Diagenetic Influences on Geochemical Proxies

Petrographic observations in both sections indicate that most samples maintain fine‐grained micritic (or dolomicritic) to microsparitic (or dolomicrosparitic) textures that preserve depositional fabrics (Figures S1–S5). Finely‐crystalline dolomite with abundant evidence of evaporite mineral growth characterizes the Staraya Rechka and Izluchina formations of the Anabar and Igarka uplifts, respectively. These observations are consistent with their accumulation in shallow peritidal to supratidal environments. In contrast, micritic limestone dominates the Manykai and Krasny Porog formations along the Kotuj and Sukharikha rivers, while mixed limestone and dolomitic limestones characterize the Sukharikha Formation carbonates. In this formation, partial dolomitization is recorded in the stratigraphic intervals associated with carbon cycle oscillations 2n–4p and 5p–6p, but does not appear to impact the fidelity of the Fortunian δ^13^C oscillations recorded in equivalent successions worldwide (Maloof et al. 2010). These dolomitic intervals, however, do record more enriched ^18^O abundances relative to limestone facies. We suggest that trends in δ^18^O_carb_ values through these strata may be more related to changes in depositional environments instead of diagenetic alteration. For example, ^18^O enrichment is recorded in fine‐grained dolostones from the gypsum‐dominated Chimuka Member of the Staraya Rechka Formation of the Anabar Uplift, which would be expected insofar as evaporation concentrates the heavy oxygen isotope in residual seawater. This is likely the explanation for ^18^O enrichment in the Izluchina Formation dolostones of the Igarka Uplift as well. More negative δ^18^O compositions recorded in Sukharikha Formation limestone facies likely record more open ocean conditions as well as the authigenic growth of secondary sparry calcite. Regardless, Mn/Sr in all the Manykai, Sukharikha, and Krasny Porog limestones and dolomitic limestones is low, suggesting good overall preservation (Kaufman et al. 1992) of these Siberian carbonates.

**FIGURE 8:**
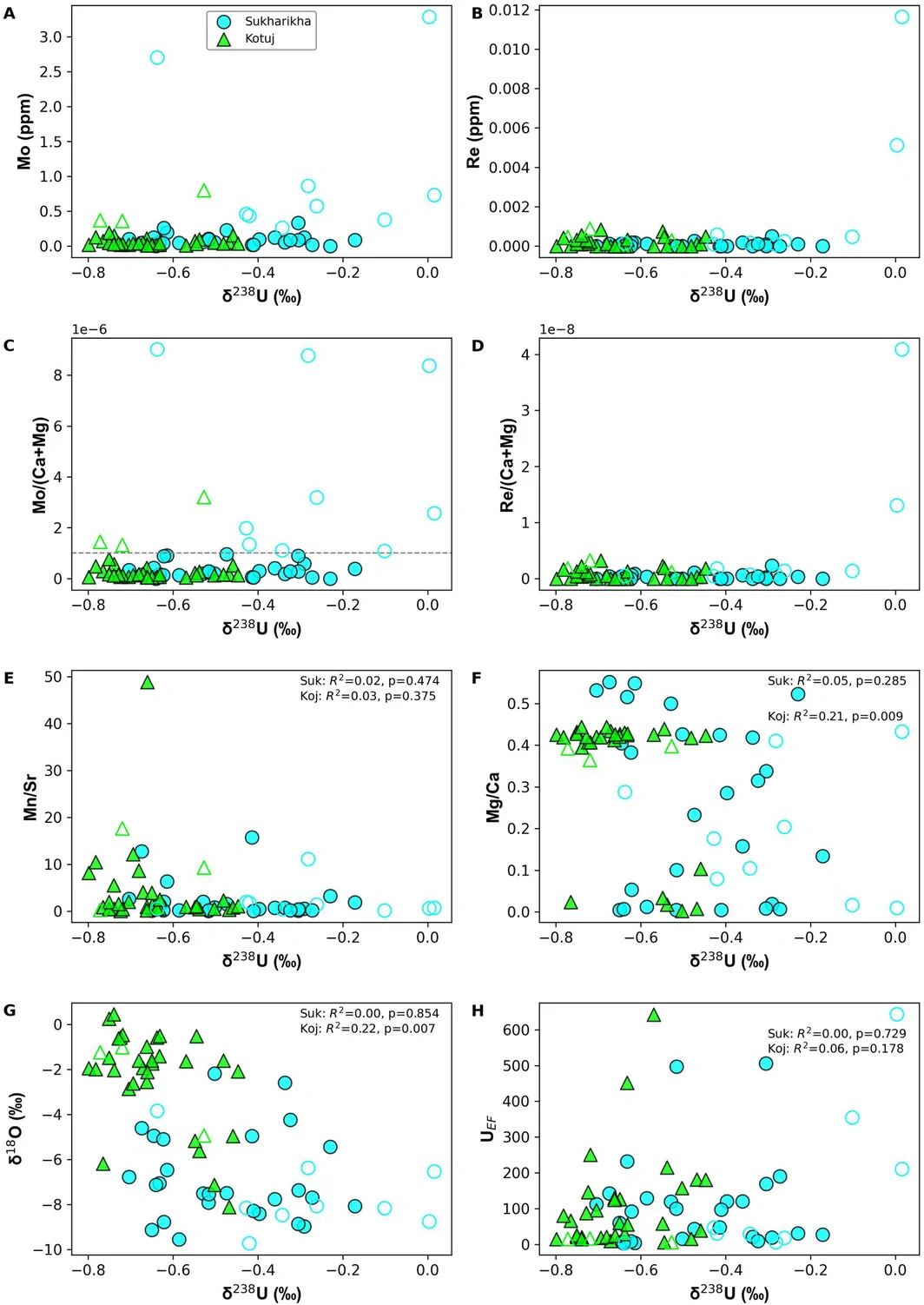
Carbonate δ^238^U vs. trace element plots for the Sukharikha River (blue circles) and Kotuj River (green triangles) sections. (A–D) δ^238^U vs. Mo, Re, Mo/(Ca + Mg), and Re/(Ca + Mg), respectively, showing some δ^238^U positive outliers at higher Mo and Re concentrations and Mo/(Ca + Mg) and Re/(Ca + Mg) ratios (open blue circles and open green triangles in all panels) that could indicate contamination with isotopically heavy U(IV) during early anoxic diagenesis. Dashed line in C = Mo/(Ca + Mg) = 1 × 10^−6^ ppm/ppm, which was the cutoff value used to potentially diagnose contamination of δ^238^U signals during early anoxic diagenesis (see text for details). (E–H) δ^238^U vs. Mn/Sr, Mg/Ca, δ^18^O, and U_EF_, respectively, along with linear correlation statistics. Suk. = Sukharikha River, Koj = Kotuj River.

Further support for the preservation of depositional isotope compositions in Sukharikha and Krasny Porog carbonates comes from our strontium isotope study of the Igarka succession (Figure 5G). The ^87^Sr/^86^Sr compositions of these samples exhibit a systematically decreasing stratigraphic trend, which broadly matches the global curve and may provide our clearest picture of strontium isotope changes through the Fortunian Stage published to date (Kaufman et al. 1996). The smooth trend is testament to the gentle extraction protocol that avoids dolomitic phases, the high strontium contents of the micrites, and the excellent degree of preservation in these samples—consistent with our detailed petrographic analysis and the low Mn/Sr of the samples. Furthermore, we find no systematic relationship between Mn/Sr, Mg/Ca, and δ^18^O compositions of the carbonates with δ^238^U values (Figure 8) suggesting that neither meteoric alteration nor dolomitization greatly impacted uranium isotope compositions, although we recognize that strong correlations between these variables are not observed in modern and Neogene carbonates that have undergone diagenetic alteration (Chen et al. 2018). Our calculations of elevated uranium enrichment factors (Figures 4F and 5F) in these samples indicate that the element is dominantly authigenic and thus derived from seawater as opposed to being detrital in origin (> 10 in nearly all samples compared to a value of 1 if all U in the sample was detrital). We further tested the relationship between δ^238^U values and the concentration of redox‐sensitive trace metals like Mo and Re insofar as the addition of isotopically heavy U(IV) to sediments under sulfidic pore water conditions may be accompanied by redox‐sensitive metal enrichments (Romaniello et al. 2016). In the Sukharikha River section, we found a clear correlation between positive outliers in δ^238^U and enrichment in Mo and Re (Figure 8A–B). This relationship is most apparent for samples with > 0.5 ppm Mo and > 1 × 10^−6^ Mo/(Ca + Mg), with no relationship seen between δ^238^U and these variables below these threshold values (see also Gilleaudeau et al. 2019). In our temporal reconstruction of δ^238^U seawater composition based on Sukharikha River samples, we thus reject samples with > 0.5 ppm Mo and > 1 × 10^−6^ Mo/(Ca + Mg). Based on these criteria, nine samples were rejected, including many of the positive δ^238^U outliers, as well as several of the higher δ^238^U values recorded across the BACE interval (Figure 7). In contrast, the Kotuj River section exhibits much lower concentrations of Mo and Re with no obvious positive δ^238^U outliers in the dataset (Figure 8) so only one sample was excluded based on the trace element criteria (Figure 6).

With respect to REE abundances, Y/Ho ratios are < 36 for a majority of samples from both the Kotuj River and Sukharikha sections (Figures 4 and 5), potentially indicating some degree of detrital contamination of the REE signals despite the gentle leaching protocol designed to avoid siliciclastic components. Nonetheless, two samples from the Sukharikha River section and six samples from the Kotuj River section yielded Y/Ho > 36, giving us a glimpse of seawater‐derived REE patterns in both sections. Wallace et al. (2017) also suggested that low Y/Ho ratios can result from anoxic depositional conditions and be unrelated to siliciclastic contamination, implying that many of our samples may be recording seawater‐derived REE patterns despite Y/Ho < 36.

### 6.2 Effect of Evaporative Conditions on Seawater δ^34^S and δ^238^U Reconstructions

Given the presence of BACE evaporite deposits on multiple continents, it is reasonable to consider whether the flux of sulfate out of the ocean had an impact on the isotopic composition of seawater proxies. In this regard, the profound upsection ^34^S enrichment of pyrite residues recorded in the evaporite‐dominated Staraya Rechka Formation (Figure 3G) provides a window to understanding the “Yudomski” event (Holser 1977) through the progressive distillation of sulfate from seawater (Okubo et al. 2022) into gypsum and anhydrite. This process would further decrease the concentration of the oxidants (in addition to O_2_) necessary for organic carbon remineralization and potentially allow for a proportionally larger fraction of chemoautotrophic inputs to the sedimentary archive—which might be recorded in the more ^13^C‐depleted organic matter in the Staraya Rechka samples. Above the Staraya Rechka Formation, the profound oscillations in Manykai sulfur isotope variations demonstrate facies control of δ^34^S compositions that were likely exacerbated by generally low Fortunian marine sulfate abundance (*contra* Canfield and Farquhar 2009; Hantsoo et al. 2018). The over 50‰ range of pyrite δ^34^S compositions is remarkable, but high‐resolution sampling notably reveals coupled oscillations of carbonate δ^13^C and pyrite δ^34^S compositions. In this regard, Manykai Members III, V, and IX, which are characterized by thrombolite and/or calcimicrobe facies, define negative excursions in both δ^13^C_carb_ and δ^34^S_pyrite_. This observation suggests the possibility of a biological control on the magnitude of both sulfur and carbon isotopic fractionation. Insofar as the oscillating secular trend in carbon isotopes is demonstrated as a global phenomenon (Maloof et al. 2010), it seems possible that the negative Fortunian δ^13^C excursions might reflect a greater authigenic component in carbonates (Schrag et al. 2013) during these events associated with the buildup of carbonate saturation through anaerobic microbial sulfate reduction (Cui et al. 2017).

In this context, we consider whether the small difference in the median uranium isotope composition of our BACE samples from the Anabar and Igarka uplifts might be explained by the dominance of evaporites in the Staraya Rechka Formation in the Anabar Uplift and the more open marine basal Sukharikha Formation in the Igarka Uplift some 750 km distant (Bowyer et al. 2023), although we recognize that uncertainties in correlation of the two sections may also explain these differences. To understand these differences, we explore how the magnitude of uranium isotope fractionation in carbonates may be impacted by seawater chemistry, especially salinity. Data from modern and Neogene carbonates of the Bahamas and South China Sea indicate a δ^238^U offset of ~0.24‰ to ~0.27‰ from seawater values, with this offset then generally applied to the uranium isotope composition of ancient carbonates to reconstruct seawater δ^238^U compositions (Chen et al. 2018; Tissot et al. 2018; Xiong et al. 2025). The additional enrichment of ^238^U in sedimentary carbonates is based on the observation that syndepositional and post‐depositional carbonates often incorporate some isotopically heavy U(IV) during early seafloor diagenesis, although the degree of δ^238^U offset between carbonates and seawater has been shown to be broadly consistent across a range of diagenetic conditions, mineralogies, and burial depths (Chen et al. 2018; Tissot et al. 2018; Xiong et al. 2025). In this study, we use a 0.24‰ offset as a baseline for reconstructing seawater δ^238^U from carbonate values across the BACE. When using this method, the Sukharikha River and Kotuj River sections exhibit median reconstructed seawater δ^238^U compositions of −0.74‰ and −0.90‰, respectively. These values are substantially lower than the modern seawater value of −0.38‰ (Kipp and Tissot 2022), indicating widespread global ocean anoxia across the BACE interval, similar to the extent of ocean anoxia recorded by U isotopes in preceding terminal Ediacaran carbonates (Zhang et al. 2018; Cherry et al. 2022).

The 0.16‰ average difference in BACE δ^238^U values between the two coeval sections, however, suggests that applying a blanket 0.24‰ offset between carbonates and seawater may not fully capture depositional and diagenetic differences that may exist between the two sections. In particular, the Kotuj River section stands out as being rich in sulfate evaporites, suggesting deposition under highly evaporative conditions, whereas the coeval Sukharikha River section mostly lacks evaporites and was likely deposited under normal marine salinities. This spatial difference in salinity across the Siberian Platform is consistent with the environmental reconstruction of the Siberian Platform proposed by Bowyer et al. (2023). Based on aqueous speciation models, Chen et al. (2017) reported that with increasing salinity (i.e., ionic strength),11 the δ^238^U fractionation between seawater and carbonate would diminish to as low as 0.06‰, compared to a fractionation factor of 0.20‰ or greater under normal marine salinities. Thus, if we apply a 0.24‰ offset between carbonate and seawater for the normal marine Sukharikha River section but a 0.06‰ offset for the highly evaporative Kotuj River section, we reconstruct a nearly identical seawater δ^238^U composition from the two sections (medians of −0.74‰ and −0.72‰ for the Sukharikha River and Kotuj River sections, respectively) indicating widespread marine anoxia across the BACE, and also supporting the stratigraphic equivalency of these sections.

It is also important to consider that this salinity effect may have only been present during some but not all of the depositional interval of the Kotuj River section. Specifically, sulfate evaporites are abundant in the Chimuka Member of the Staraya Rechka Formation, but are largely absent in the Kharyyalakh and Kochokon members and the overlying Manykai Formation. Based on these observations, we also reconstructed seawater δ^238^U values by applying a smaller offset of 0.06‰ only to samples from the Chimuka Member, with a 0.24‰ offset applied to the rest of the samples from the Kotuj River and Sukharikha River sections (Figures 7 and 8). Under this scenario, the Kotuj River section begins with a median reconstructed seawater δ^238^U value of −0.93‰ for the basal Kharyyalakh Member and then rises to a median value of −0.77‰ for the Chimuka Member. Seawater δ^238^U then falls back to a median of −0.81‰ in the overlying Kochokon Member and Manykai Formation that records the post‐BACE Fortunian interval (Figure 6C). This suggests a subtle rise in seawater δ^238^U across the BACE in the Kotuj River section, which is consistent with a similar transient δ^238^U rise recorded across the BACE in the Sukharikha River section (Figure 7C), although in the Igarka Uplift this rise is only recorded by four data points over a relatively narrow stratigraphic range. Overall, we suggest that it is important to consider the effects of salinity and ionic strength on the fractionation factor between seawater and carbonates when strata show sedimentological evidence for evaporative depositional conditions.

### 6.3 Global Ocean Redox Across the BACE

**FIGURE 9:**
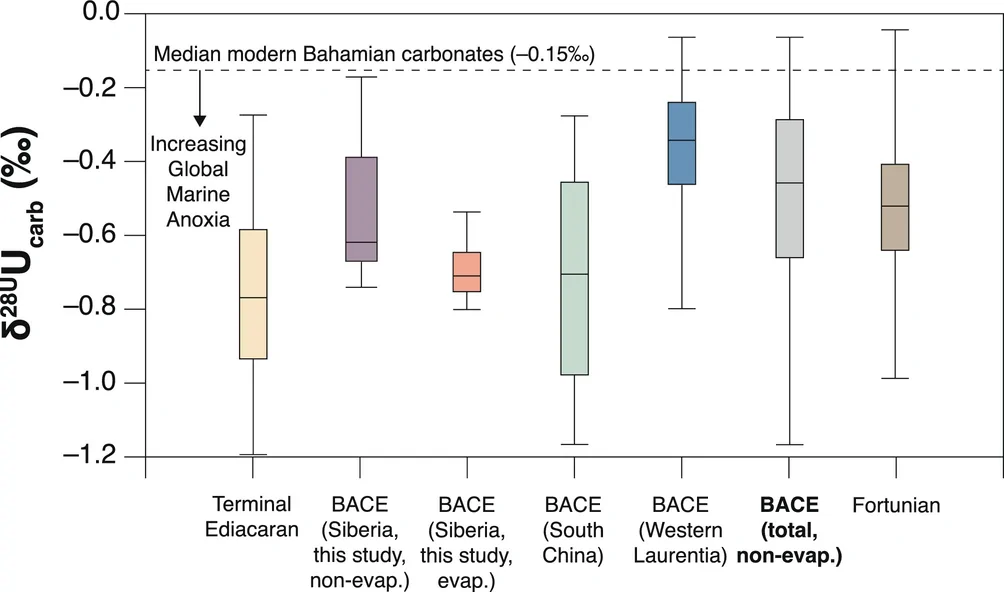
Global compilation of carbonate δ^238^U data for the terminal Ediacaran, BACE, and Fortunian intervals. The terminal Ediacaran encompasses data (*n* = 160) from South China (Zhang et al. 2018), Namibia (Tostevin et al. 2019), and Siberia (Cherry et al. 2022), as well as additional select data from South China (Wei et al. 2018) constrained as older than 541 Ma based on the age model of Bowyer et al. (2024). Non‐evaporative (non‐evap.) BACE samples from this study (both Sukharikha River and Kotuj River sections, *n* = 16) are shown alongside evaporative (evap.) BACE samples from the Kotuj River, this study (*n* = 15). Samples that were excluded from this study based on trace element screening criteria are not shown. BACE data from South China (*n* = 11) are select samples from Wei et al. (2018) constrained between 541 and 533 Ma based on the age model of Bowyer et al. (2024). BACE samples from Western Laurentia (*n* = 41) are from the Mount Dunfee section (USA) and Cerro Rajón section (Mexico) from Chanchai et al. (2026). The total of non‐evaporative BACE samples encompasses all BACE samples described above except the evaporative interval of the Kotuj River section (this study), as well as three samples from Siberia reported by Dahl et al. (2019) that are constrained as older than 533 Ma based on the age model of Bowyer et al. (2024). The Fortunian encompasses data (*n* = 123) from the post‐BACE intervals of the Sukharikha River and Kotuj River sections (this study) and data from South China, Siberia, and Morrocco (Wei et al. 2018; Dahl et al. 2019) constrained between 533 and 529 Ma based on the age model of Bowyer et al. (2024). Box‐and‐whisker plots display minimum, 25th percentile, median, 75th percentile, and maximum δ^238^U values. Three datapoints with δ^238^U > 0‰ were excluded. The median δ^238^U value of modern Bahamian carbonates (−0.15‰) is from Chen et al. (2018).

Based on the information summarized above, we conclude that the BACE was only characterized by relatively limited and transient ocean oxygenation, with the global oceans remaining significantly more anoxic than today through the entire BACE and subsequent Fortunian and Cambrian Stage II interval. This is evidenced by carbonate and reconstructed seawater δ^238^U values that remain well below the baseline of the modern well‐oxygenated ocean throughout both of our studied sections. The low δ^238^U values across the BACE appear as a continuation of redox conditions recorded in terminal Ediacaran carbonates from Siberia (Cherry et al. 2022), South China (Zhang et al. 2018), and Namibia (Tostevin et al. 2019). We also recognize that uncertainties in stratigraphic correlation imply that our δ^238^U data from the Kotuj River negative δ^13^C excursion may be older than the BACE, and thus also represent terminal Ediacaran deposition (Marusin et al. 2025). If this is the case, however, our δ^238^U data still imply persistent global ocean anoxia across the broad terminal Ediacaran to Early Cambrian interval. Thus, it seems that the global ocean experienced widespread anoxia in the terminal Ediacaran that persisted into the Cambrian with only a subtle increase in oxygenation across the BACE event (Figure 9). Notably, in our dataset from the Sukharikha River, low δ^238^U values persist for the entire post‐BACE Fortunian and early Cambrian Stage II interval despite fluctuating δ^13^C values, and Cherry et al. (2022) similarly reported low δ^238^U values in overlying Cambrian Stage II carbonates from the Olenek River section, Siberia. These data broadly match with intervals of low δ^238^U values seen in a compilation of Early Cambrian carbonates also from Siberia (Dahl et al. 2019). Thus, the combined δ^238^U record suggests that widespread global ocean anoxia was persistent through the terminal Ediacaran, Fortunian, and Cambrian Stage II, with only limited oxygenation recorded across the BACE (Figure 9).

Our new δ^238^U record is broadly consistent with Chanchai et al. (2026), who recently reported δ^238^U values from two carbonate sections across the BACE interval in southwestern Laurentia. There is no systematic change in δ^238^U recorded across the BACE interval in either the Mount Dunfee section (USA) or Cerro Rajón section (Mexico), although carbonate δ^238^U values are generally higher in these sections compared to our Siberian sections (Figure 9). In both cases, however, the δ^238^U values are notably lower than modern carbonates deposited in a well‐oxygenated global ocean, indicating widespread global ocean anoxia across the BACE interval (Figure 9). Interestingly, Ce/Ce* data from these two southwestern Laurentia sections show values largely consistent with local anoxia (Ce/Ce* > 0.9) except for a transient local oxygenation at one of the two sections (Cerro Rajón). In contrast, all eight samples from our study of the Sukharikha and Kotuj River sections with Y/Ho > 36 exhibit Ce/Ce* > 0.9, indicating locally anoxic conditions. In addition, if we consider that samples with lower Y/Ho ratios may also be recording seawater REE signatures (Wallace et al. 2017), then the large majority of our dataset suggests locally anoxic conditions (64 of 72 total samples have Ce/Ce* > 0.9). Locally anoxic conditions are particularly relevant in the Kotuj River section (where all samples have Ce/Ce* > 0.9) because it was deposited in shallow, nearshore evaporative environments, suggesting that anoxia extended into shallow settings in a generally low‐O_2_ ocean–atmosphere system. Overall, it is apparent that transient local oxygenation may have occurred in some but not all shallow marine settings across the BACE interval.

Some oxygenation across the BACE interval is also supported by δ^238^U data from the Gaojiaxi‐Yanjiahe and Xiaotan sections in South China, where δ^238^U values rise from a low baseline of ~−1.0‰ in the terminal Ediacaran to ~−0.5‰ associated with negative δ^13^C values broadly across the Ediacaran‐Cambrian transition (Wei et al. 2018). This δ^238^U shift occurs in only a few data points and in mixed facies, however, including cherty dolostone and phosphatic dolostone, and the interval of the Ediacaran–Cambrian boundary is stratigraphically condensed and contains a covered (likely siliciclastic‐rich) interval in the Xiaotan section. Despite these complications, the δ^238^U signal from these South China sections is broadly consistent with our non‐evaporative Sukharikha River section that shows some ^238^U enrichment through the BACE interval, but not to the level of modern carbonates. In addition to the four somewhat ^238^U enriched samples recorded across the BACE in the Sukharikha River section, three data points higher in the same section record a second transient rise in δ^238^U that could be interpreted as a short‐lived oxygenation event. These data points seem to correspond to negative δ^13^C excursion 3n in the middle Fortunian and, like the BACE, δ^238^U values do not rise to the level of modern carbonates (Figure 7).

Overall, our δ^238^U results are consistent with the global compilation of shale iron speciation (Sperling et al. 2015) and trace metal concentration data from the Sedimentary Geochemistry and Paleoenvironments Database (Stockey et al. 2024), which collectively argue against widespread oxygenation of the oceans across the Ediacaran–Cambrian transition. Based on rising shale TOC contents and cGENIE modeling results, Stockey et al. (2024) suggested a subtle rise in marine primary productivity, atmospheric *p*O_2_, and shallow ocean O_2_ concentrations across the Ediacaran–Cambrian transition, while much of the deep oceans remained anoxic and total anoxic seafloor area remained > 10%, which is largely unchanged from the earlier Proterozoic. This rise in atmospheric and shallow ocean O_2_ levels could have been driven in part by tectonic reorganization and the onset of the terrestrial “clay mineral factory” across the Ediacaran–Cambrian transition, which promoted enhanced organic carbon burial through clay‐organic complexation (Kennedy et al. 2006; Wei et al. 2024; Gan et al. in revision). This hypothesis is supported by δ^7^Li data indicating a strongly incongruent continental weathering regime across the Ediacaran‐Cambrian transition as recorded in the same Siberian carbonates examined in this study (Gan et al., in revision). Despite evidence for subtle oxygenation, studies of subsequent Early Paleozoic ocean redox based on shale iron speciation and trace metal concentration compilations, carbonate Ce anomalies and I/Ca data, as well as Mo, U, and S isotopes in a variety of shale and carbonate strata, collectively suggest that widespread global ocean anoxia persisted well into the Paleozoic (Dahl et al. 2010; Gill et al. 2011; Wallace et al. 2017; Lu et al. 2018; Haxen et al. 2023; Stacey et al. 2024; Stockey et al. 2024; Alexander et al. 2025). Large‐scale oxygenation of the oceans to modern levels likely only occurred beginning in the Devonian Period associated with the proliferation of rooted land plants (Dahl et al. 2010; Lenton et al. 2016; Elrick et al. 2022; Stockey et al. 2024; Ostrander et al. 2025).

### 6.4 Implications for the Extinction of the Ediacara Biota

Overall, our δ^238^U data suggest that widespread and sustained ocean oxygenation was not responsible for the extinction of the Ediacara biota, which appear to have been well adapted to low‐oxygen conditions (Kaufman et al. 2019; Hammarlund 2020; Cherry et al. 2022). Instead, our data suggest that the low‐oxygen oceans in which the Ediacara biota thrived persisted well into the Cambrian, with only limited and local ocean oxygenation recorded across the BACE. We conclude that a major change in whole ocean redox did not likely cause the profound evolutionary and ecological restructuring that occurred across the Ediacaran–Cambrian transition. A local increase in shallow ocean O_2_ concentrations could still have plausibly contributed to the demise of the Nama assemblage that dominated the interval immediately before the BACE insofar as these organisms inhabited mostly shallow shelf environments where fluctuating redox conditions have been interpreted from iron speciation data (Wood et al. 2015, 2019). This contrasts, however, with the dominantly anoxic Ce/Ce* signal recorded in strata from this study, although the strong detrital influence on REE patterns (Y/Ho < 36) potentially precludes detailed time‐series comparisons. Increasing shelf O_2_ levels could have favored the more energetic and O_2_‐demanding organisms of the Cambrian fauna, to the detriment of the Ediacara biota that may have exploited redox gradients in low‐oxygen or hypoxic oceans. Furthermore, Sperling et al. (2013) proposed a link between oxygen and the biotic replacement model (Laflamme et al. 2013), arguing that locally rising O_2_ levels (even in a low‐O_2_ world) could lead to an increased ecological role for predators, which would then in turn drive the predator–prey “arms races” that led to competitive exclusion of the Ediacara biota and, eventually, the Cambrian animal radiation.

Our interpretation of global redox conditions across the BACE suggests that other factors beyond ocean oxygenation played a critical role in the extinction of the Ediacara biota and their replacement with Cambrian animals. As suggested by the abundant evaporites present in the Kotuj River section BACE interval (with other examples in Siberia including the Platonovskaya Formation in the Turukhansk Uplift; Bartley et al. 1998; Marusin et al. 2022, and the Turkut Formation in the Olenek Uplift; Grazhdankin et al. 2020) and throughout the Middle East, Pakistan, India, and China, the BACE interval was likely associated with sea‐level fall and strongly evaporative conditions on shallow shelves and in restricted basins. Thus, global exposure of continental shelves and the concomitant increase in seawater salinity would have been detrimental to the Ediacaran biota that were largely immobile, had high surface areas, and relied heavily on diffusion (Dufour and McIlroy 2017). Insofar as the biota were simply bags of water, subaerial exposure and/or dehydration through osmosis—at least in nearshore environments—could have been an important kill mechanism for these early experiments in multicellularity. In addition, sea‐level fall could have exposed Ediacaran organisms to large diurnal and seasonal temperature changes in shallow water, moving them away from the stenothermal environments which may have promoted their initial diversification (Boag et al. 2018).

## 7 Conclusions

Here, we present new δ^238^U and Ce/Ce* data across the BAsal Cambrian carbon isotope Excursion (BACE) from two carbonate sections on the Siberian Platform (Kotuj River and Sukharikha River), new ^87^Sr/^86^Sr data from the Sukharikha River section that adds to the global ^87^Sr/^86^Sr database of the BACE and Fortunian intervals, and new δ^34^S data from a third Siberian carbonate section (Kotujkan River). When reconstructing seawater δ^238^U from carbonate values, we considered the effect of strongly evaporative depositional conditions in the Chimuka Member of the Kotuj River section, which may have muted the isotopic offset between carbonate and seawater in this interval compared to the normal marine conditions that prevailed for most of the rest of the Kotuj River and Sukharikha River sections. When considering this salinity effect, the median reconstructed seawater δ^238^U value is −0.74‰ and −0.79‰ for the Sukharikha River and Kotuj River sections, respectively—substantially lower than the modern seawater value, indicating expansive global ocean anoxia across the Ediacaran–Cambrian transition. Specifically across the BACE, there is only a subtle and transient rise in δ^238^U values, with reconstructed seawater δ^238^U remaining less than −0.60‰ except for four data points in the Sukharikha River section that reach a median δ^238^U value of −0.54‰. These persistently low δ^238^U values represent a continuation of similarly low values recorded globally in the terminal Ediacaran (Zhang et al. 2018; Tostevin et al. 2019; Cherry et al. 2022) and match a new BACE δ^238^U record from southwestern Laurentia (Chanchai et al. 2026).

Overall, our results indicate that the Ediacaran–Cambrian transition did not experience a large and unidirectional rise in oceanic O_2_ levels. Ocean oxygenation across the BACE did not reach the level of modern oceans, and it was only transient, with widespread anoxia (similar in magnitude to the terminal Ediacaran) persisting through the Fortunian and Cambrian Stage II. Thus, we suggest that widespread ocean oxygenation was not the ultimate cause of the demise of the Ediacara biota, which may have been well‐adapted to life under low‐oxygen or hypoxic conditions (Hammarlund 2020; Cherry et al. 2022). If seawater oxygenation did rise locally in shallow marine environments, our data might still support the biotic replacement model where the advent of predation and ecosystem engineering caused the Ediacara biota to be outcompeted by Cambrian faunas. Subtle rises in atmospheric *p*O_2_ and shallow shelf O_2_ concentrations (Stockey et al. 2024)—potentially driven by increased terrestrial clay production that promoted organic carbon burial (Gan et al., in revision)—could have favored the more energetic and O_2_‐demanding organisms of the Cambrian fauna, to the detriment of the Ediacara biota, in oceans that remained substantially less oxygenated than today.

## Funding

Geoffrey J. Gilleaudeau and Alan J. Kaufman are grateful for financial support from the National Science Foundation Geobiology and Low‐Temperature Geochemistry Program. Geoffrey J. Gilleaudeau additionally thanks the American Chemical Society Petroleum Research Fund and the NASA Exobiology Program for financial support.

## Conflicts of Interest

The authors declare no conflicts of interest.

## Supporting information


**Figure S1:** Outcrop and petrographic observations of the Staraya Rechka Formation in the southern Anabar Uplift along the Kotuj River. (A) Kharyylakh Member at ~11 m above the base of the Staraya Rechka Formation characterized by thin to medium bedded dolostone domal stromatolites with abundant vugs formed from the dissolution of evaporite minerals; (B) sample KtB‐241/1 from location described in A with blue epoxy filling voids of former anhydrite or halite crystals embedded in a fine‐grained dolomicrospar; (C) Chimuka Member at ~30 m height thin bedded dolostone lens amid thick nodular (chicken wire) anhydrite; (D) sample KtB‐232/5 from location described in C with subrounded anhydrite nodules or clotted fabric of potential calcareous algae; (E) thin evaporites alternating with medium bedded vuggy dolostone from ~71 m in the Chimuka Member; (F) mixed dolomicrospar, coarse grained anhydrite, and potential fragments of amorphous algal sheets or microbial mats in sample KtB‐246/10 from location described in E; (G) Kochokon Member at ~81 m with thin to medium bedded vuggy dolostone; (H) sample KtB‐233/17 from location described in G with dolomicrospar matrix and calcareous algae similar to *Epiphyton* or *Renalcis*.
**Figure S2:** Outcrop and petrographic observations of the Manykai Formation in the southern Anabar Uplift along the Kotuj River. (A) Manykai outcrop ~166 m above the base of the succession of medium bedded limestone grainstone; (B) sample KtB‐249/36 from location described in A showing a laminated limestone grainstone including small rounded peloidal grains and secondary sparry cement along with ubiquitous hematite (likely after pyrite) in the matrix and concentrated at laminations along with occasional needles of evaporite minerals; (C) outcrop at ~174 m of limestone grainstone with large vugs of secondary recrystallized calcite potentially replacement after evaporite minerals; (D) sample KtB‐249/45 from location described in C of large (300–400 μm diameter) peloids of mixed microspar and sparite in a sparry matrix; (E) sugary limestone at ~194 m from uppermost stromatolitic reef complex at top of measured succession; (F) sample KtB‐246/77 from the location described in E of mixed microspar and sparite along with minor hematite (likely after pyrite); (G) surface expression of synaeresis cracks at ~27 m above the formation base; (H) lozenge‐shaped vugs after gypsum dissolution at ~40 m above the formation base.
**Figure S3:** Outcrop observations of trace and body fossils the Manykai Formation in the southern Anabar Uplift along the Kotuj River. (A) *Torrowangea* and cross sections of *Skolithos*, positive hyporelief, ~25 m above the formation base; (B) straight to sinuous *Palaeophycus* and short straight synaeresis cracks, positive hyporelief, 35 m above the formation base; (C) *Didymaulichnus* and short *Palaeophycus*, negative epirelief, 27 m above the formation base; (D) straight to sinuous *Palaeophycus*, positive hyporelief, 35 m above the formation base; (E) straight to arcuate *Palaeophycus* and synaeresis cracks, ~25 m above the formation base; (F) synaeresis cracks, ~25 m above the formation base; (G) *Thalassinoides* (?) boxworks, positive hyporelief, or sponge‐grade animals, ~40 m above the formation base; (H) possible archeocyathids, ~42 m above the formation base.
**Figure S4:** Outcrop and petrographic observations of the Izluchin and Sukarihka formations in the Igarka Uplift along the Sukharikha River. (A) Izluchin outcrop ~55 m above the base of the measured succession of peritidal dolomitic microbialaminite including fibrous precipitate fabrics suggestive of high carbonate saturation and replacement of bushy mounded structures with iron and silica; (B) sample U1801‐8 from location described in A showing clotted microbial fabric with potential microbial or algal sheets and potential evaporite‐related vug; (C) outcrop at ~84 m of the Izluchin/Sukharikha transition with interbedded red to gray thinly laminated siltstones alternating with medium bedded dolomitic microbialaminite; (D) sample U1801‐26 four meters above the base of the Sukharikha Formation of clotted dolomite grainstone (?) or microbial (?) fabric with carbonate cement; (E) Sukharikha outcrop at ~172 m of domal limestone stromatolites; (F) sample U1801‐44 of clotted limestone grainstone (?) or microbial (?) fabric with carbonate cement; (G) Sukharikha outcrop at ~230 m of fine‐grained limestone micrite preferentially dolomitized along bedding surfaces; (H) sample U1801‐54 of coarse recrystallized limestone with dolomite rhombs.
**Figure S5:** Outcrop and petrographic observations of the Sukharika and Krasny Porog formations in the Igarka Uplift along the Sukharikha River. (A) Sukharikha limestone outcrop ~496 m above the base of the measured section with polygonal desiccation cracks indicative of episodic exposure; (B) sample U1801‐100 from the interval illustrated in A with coarse micro‐breccia with calcite cement and occasional dolomite rhombs; (C) tilted bedding of limestone and dolostone from the upper Sukharikha Formation at ~570 m; (D) sample U1801‐109 of recrystallized limestone and coarse euhedral dolomite from the interval described in C; (E) the gradual transition at ~650 m across the Sukharikha/Krasny Porog boundary from grey limestone of the former to pink or red silt intensely bioturbated limestone of the latter; (F) fine‐grained micrite with coarsely recrystallized fossil fragments; (G) red silty limestone of the Krasny Porog Formation at ~660 m near the top of the measured succession; (H) sample U1801‐152 from the outcrop in G showing an archaeocyathid fragment surrounded by *Epiphyton* calcareous algae and coarse infilling calcite cement.

**Data S1:** gbi70066‐sup‐0001‐DataS1.xlsx.

## Data Availability

The data that support the findings of this study are available in the Supporting Information of this article.
