# Supplementary material for: Uranium Isotope Evidence for Persistent Global Ocean Anoxia Across the Ediacaran–Cambrian Transition in Siberia
Source: Geobiology. 2026 Sep 26;24(5):e70066. doi: 10.1111/gbi.70066 (PMC13615737; doi:10.1111/gbi.70066)
Supplement: Supplementary file 1 [file GBI-24-e70066-s002.pdf]

Supplementary Information for

Uranium isotope evidence for persistent global ocean anoxia across the Ediacaran–Cambrian transition in Siberia

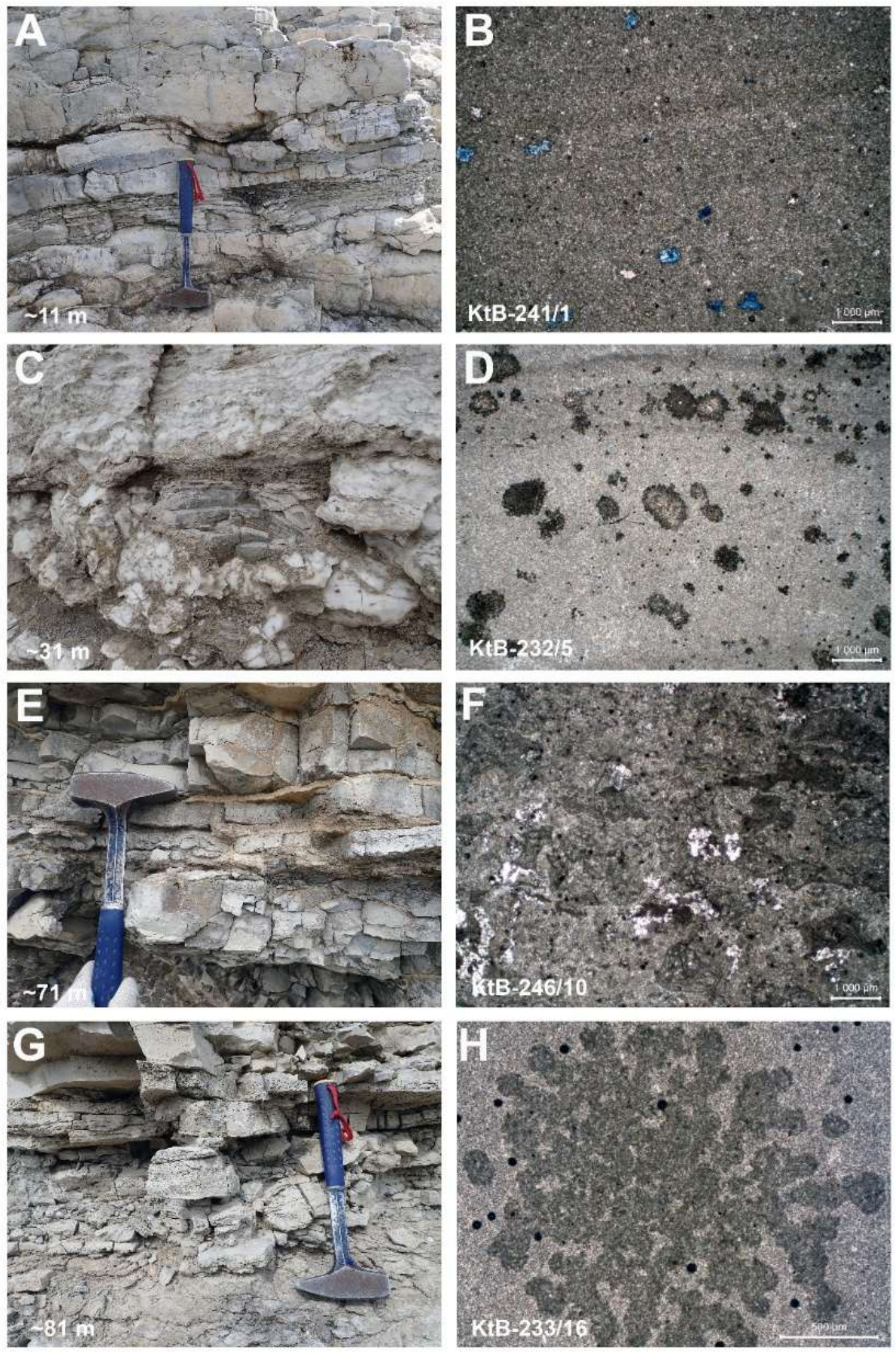

FIGURE S1 | Outcrop and petrographic observations of the Staraya Rechka Formation in the southern Anabar Uplift along the Kotuj River. **A.** Kharyylakh Member at ~11 meters above the base of the Staraya Rechka Formation characterized by thin to medium bedded dolostone domal stromatolites with abundant vugs formed from the dissolution of evaporite minerals; **B.** sample KtB-241/1 from location described in A with blue epoxy filling voids of former anhydrite or halite crystals embedded in a fine grained dolomicrospar; **C.** Chimuka Member at ~30 meters height thin bedded dolostone lens amid thick nodular (chicken wire) anhydrite; **D.** sample KtB-232/5 from location described in C with subrounded anhydrite nodules or clotted fabric of potential calcareous algae; **E.** thin evaporites alternating with medium bedded vuggy dolostone from ~71 meters in the Chimuka Member; **F.** mixed dolomicrospar, coarse grained anhydrite, and potential fragments of amorphous algal sheets or microbial mats in sample KtB-246/10 from location described in E; **G.** Kochokon Member at ~81 meters with thin to medium bedded vuggy dolostone; **H.** sample KtB-233/17 from location described in G with dolomicrospar matrix and calcareous algae similar to *Epiphyton* or *Renalcis*.

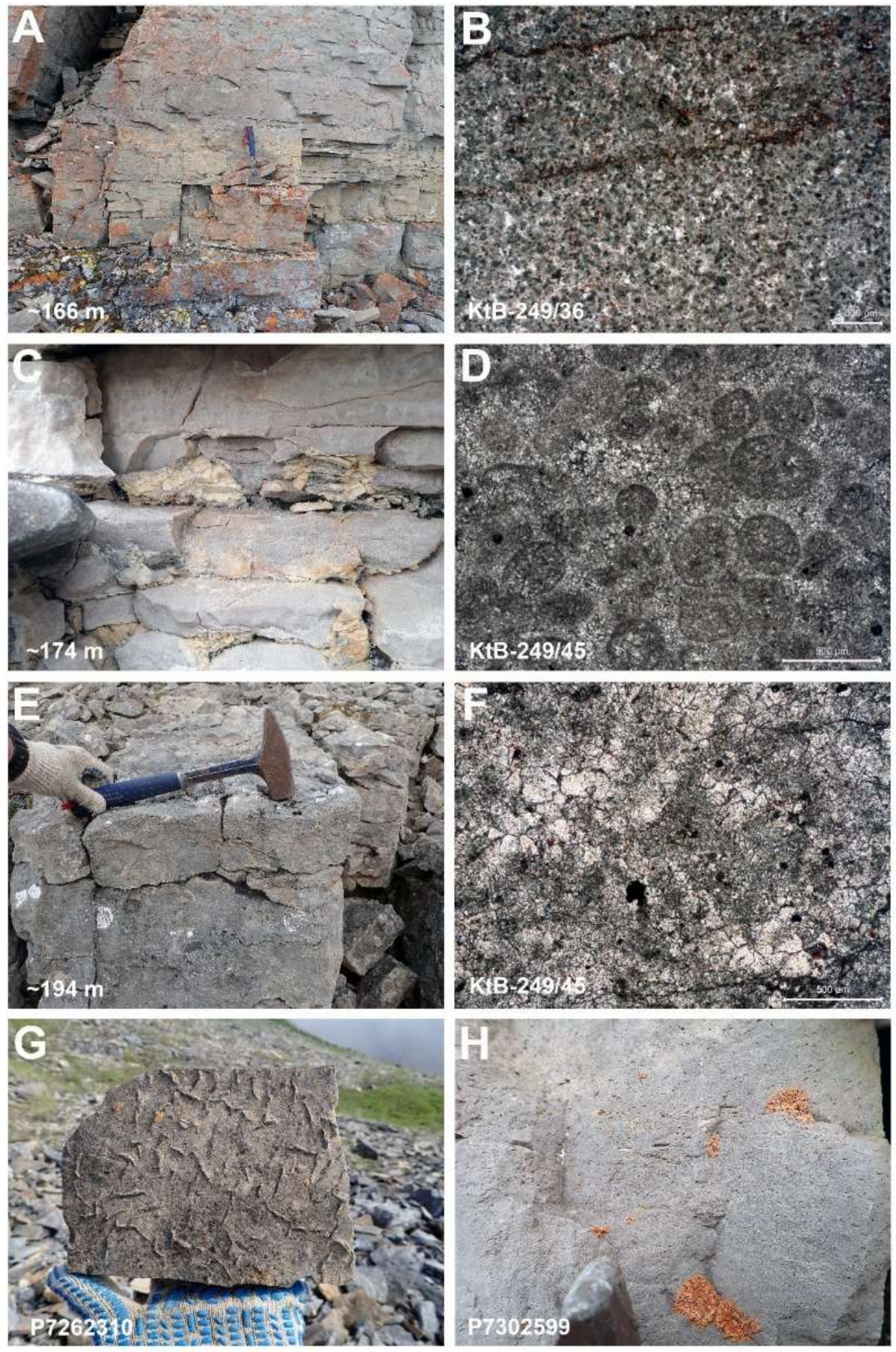

FIGURE S2 | Outcrop and petrographic observations of the Manykai Formation in the southern Anabar Uplift along the Kotuj River. **A.** Manykai outcrop ~166 meters above the base of the succession of medium bedded limestone grainstone; **B.** sample KtB-249/36 from location described in A showing a laminated limestone grainstone including small rounded peloidal grains and secondary sparry cement along with ubiquitous hematite (likely after pyrite) in the matrix and concentrated at laminations along with occasional needles of evaporite minerals; **C.** outcrop at ~174 meters of limestone grainstone with large vugs of secondary recrystallized calcite potentially replacement after evaporite minerals; **D.** sample KtB-249/45 from location described in C of large (300-400 μm diameter) peloids of mixed microspar and sparite in a sparry matrix; **E.** sugary limestone at ~194 meters from uppermost stromatolitic reef complex at top of measured succession; **F.** sample KtB-246/77 from the location described in E of mixed microspar and sparite along with minor hematite (likely after pyrite); **G.** surface expression of synaeresis cracks at ~27 m above the formation base; **H.** lozenge-shaped vugs after gypsum dissolution at ~40 m above the formation base.

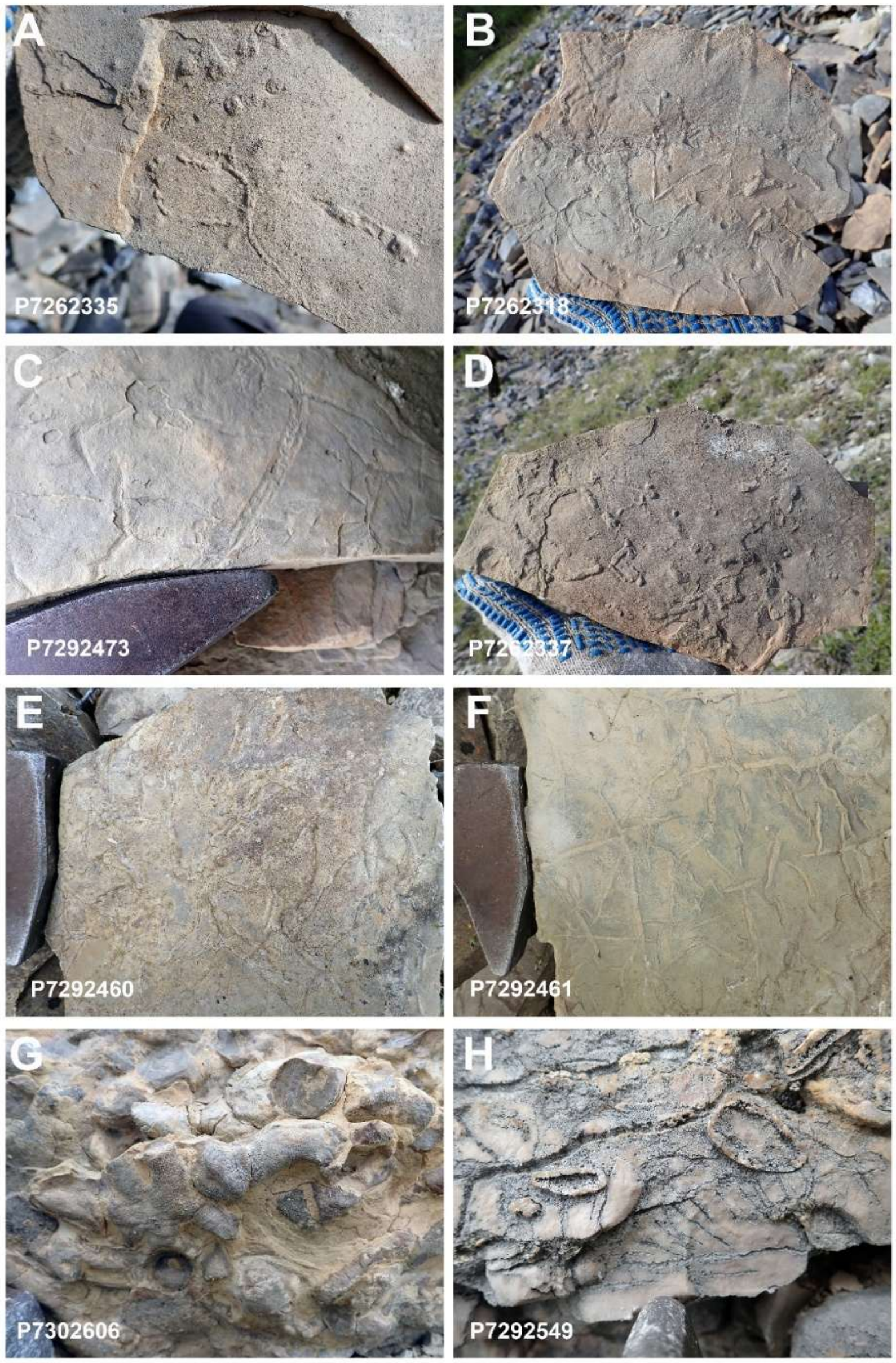

FIGURE S3 | Outcrop observations of trace and body fossils the Manykai Formation in the southern Anabar Uplift along the Kotuj River. **A.** *Torrowangea* and cross sections of *Skolithos*, positive hyporelief, ~25 m above the formation base; **B.** straight to sinuous *Palaeophycus* and short straight synaeresis cracks, positive hyporelief, 35 m above the formation base; **C.** *Didymaulichnus* and short *Palaeophycus,* negative epirelief, 27 m above the formation base; **D.** straight to sinuous *Palaeophycus*, positive hyporelief, 35 m above the formation base; **E.** straight to arcuate *Palaeophycus* and synaeresis cracks, ~25 m above the formation base; **F.** synaeresis cracks, ~25 m above the formation base; **G.** *Thalassinoides* (?) boxworks, positive hyporelief, or sponge-grade animals, ~40 m above the formation base; **H.** possible archeocyathids, ~42 m above the formation base.

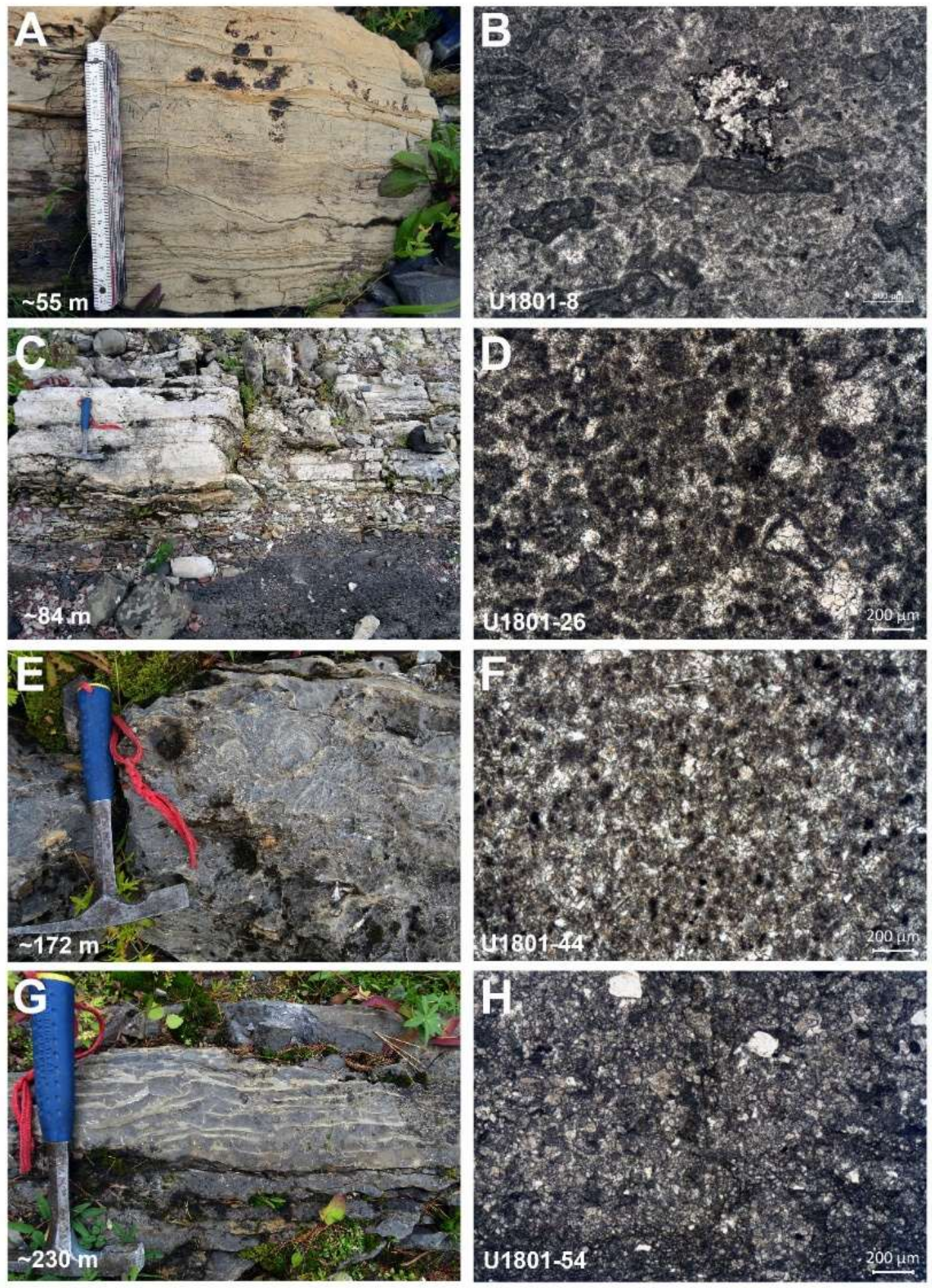

FIGURE S4 | Outcrop and petrographic observations of the Izluchin and Sukarihka formations in the Igarka Uplift along the Sukharikha River. **A.** Izluchin outcrop ~55 meters above the base of the measured succession of peritidal dolomitic microbialaminite including fibrous precipitate fabrics suggestive of high carbonate saturation and replacement of bushy mounded structures with iron and silica; **B.** sample U1801-8 from location described in A showing clotted microbial fabric with potential microbial or algal sheets and potential evaporite-related vug; **C.** outcrop at ~84 meters of the Izluchin/Sukharikha transition with interbedded red to gray thinly laminated siltstones alternating with medium bedded dolomitic microbialaminite; **D.** sample U1801-26 four meters above the base of the Sukharikha Formation of clotted dolomite grainstone (?) or microbial (?) fabric with carbonate cement; **E.** Sukharikha outcrop at ~172 meters of domal limestone stromatolites; F. sample U1801-44 of clotted limestone grainstone (?) or microbial (?) fabric with carbonate cement; **G.** Sukharikha outcrop at ~230 meters of fine-grained limestone micrite preferentially dolomitized along bedding surfaces; **H.** sample U1801-54 of coarse recrystallized limestone with dolomite rhombs.

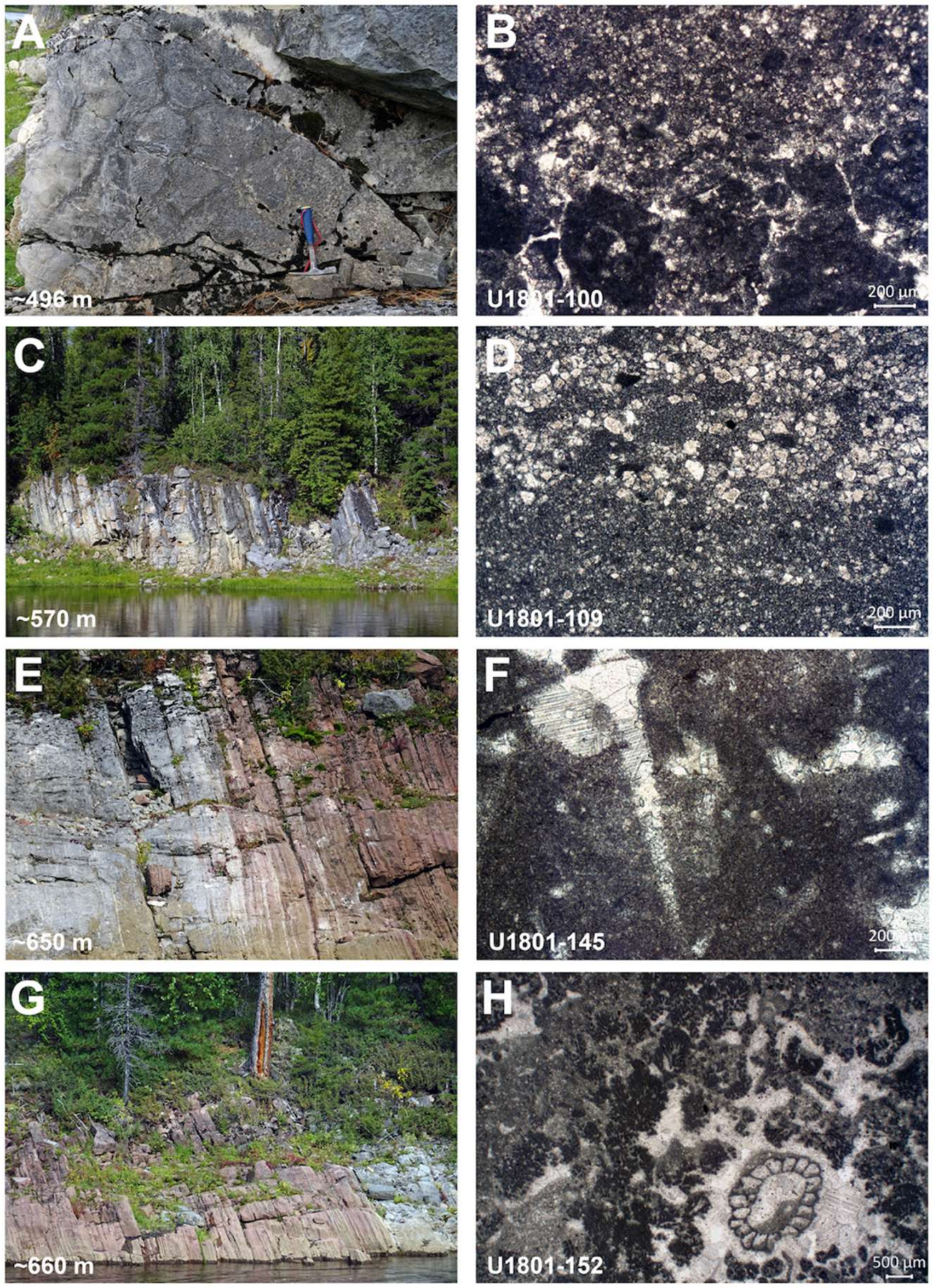

FIGURE S5 | Outcrop and petrographic observations of the Sukharika and Krasny Porog formations in the Igarka Uplift along the Sukharikha River. **A.** Sukharikha limestone outcrop ~496 meters above the base of the measured section with polygonal desiccation cracks indicative of episodic exposure; **B.** sample U1801-100 from the interval illustrated in A with coarse micro-breccia with calcite cement and occasional dolomite rhombs; **C.** tilted bedding of limestone and dolostone from the upper Sukharikha Formation at ~570 meters; **D.** sample U1801-109 of recrystallized limestone and coarse euhedral dolomite from the interval described in C; **E.** the gradual transition at ~650 meters across the Sukharikha/Krasny Porog boundary from grey limestone of the former to pink or red silty intensely bioturbated limestone of the latter; **F.** fine-grained micrite with coarsely recrystallized fossil fragments; **G.** red silty limestone of the Krasny Porog Formation at ~660 meters near the top of the measured succession; **H.** sample U1801-152 from the outcrop in G showing an archaeocyathid fragment surrounded by *Epiphyton* calcareous algae and coarse infilling calcite cement.
